# Validity and agreement between dual-energy X-ray absorptiometry, anthropometry and bioelectrical impedance in the estimation of fat mass in young adults

**DOI:** 10.3389/fnut.2024.1421950

**Published:** 2024-06-11

**Authors:** Malek Mecherques-Carini, Mario Albaladejo-Saura, Raquel Vaquero-Cristóbal, Nicolás Baglietto, Francisco Esparza-Ros

**Affiliations:** ^1^International Kinanthropometry Chair, UCAM Universidad Católica San Antonio de Murcia, Murcia, Spain; ^2^Department of Physical Activity and Sport Sciences, Faculty of Sport Sciences, University of Murcia, San Javier, Spain

**Keywords:** body composition, fat mass, anthropometry, bioelectrical impedance analysis, dual-energy X-ray absorptiometry

## Abstract

**Introduction:**

Assessment of fat mass has historically employed various methods like Dual-energy X-ray Absorptiometry (DXA), and bioelectrical impedance (BIA), and anthropometry with its set of formulas. However, doubts persist regarding their validity and interchangeability to evaluate fat mass. This research aimed to determine the validity of anthropometry, and BIA in estimating fat mass Vs DXA, considering the influence of sex and hydration status.

**Methods:**

A descriptive, cross-sectional study included 265 young adults (161 males and 104 females), assessed through DXA, BIA in a standing position, and anthropometry. A fat mass estimation formula with DXA, a fat mass estimation formula with BIA and 10 fat mass estimation formulas with anthropometry were calculated.

**Results:**

Significant differences were found across DXA, BIA and anthropometry in both kilograms and percentages for the overall sample (*p*<0.001), and when the covariable sex was included (p<0.001), with no significant effect of hydration status (*p*=0.332-0.527). Bonferroni-adjusted analyses revealed significant differences from DXA with anthropometry and BIA in most cases for the overall sample (*p*<0.001), as well as when stratified by sex (*p*<0.001–0.016). Lin’s coefficient indicated poor agreement between most of the formulas and methods both in percentage and kilograms of fat mass (CCC=0.135–0.892). In the Bland-Altman analysis, using the DXA fat mass values as a reference, lack of agreement was found in the general sample (*p*<0.001-0.007), except for Carter’s formula in kilograms (*p*=0.136) and percentage (*p*=0.929) and Forsyth for percentage (*p*=0.365). When separating the sample by sex, lack of agreement was found in males for all methods when compared with both percentage and kilograms calculated by DXA (*p*<0.001). In the female sample, all methods and formulas showed lack of agreement (*p*<0.001–0.020), except for Evans’s in percentage (*p*=0.058).

**Conclusion:**

The formulas for fat mass assessment with anthropometry and BIA may not be valid with respect to the values reported with DXA, with the exception of Carter’s anthropometry formula for general sample and Evans’s anthropometry formula for female sample. BIA could also be an alternative if what is needed is to assess fat mass in women as a group.

## 1 Introduction

The importance of fat mass in different areas is clear (1–3). In the health field, fat levels have been associated with different pathologies and comorbidities, both due to the risk of developing them and the loss of daily functionality resulting from an excess of fat mass (4). Additionally, estimating fat mass has garnered significant interest in the field of sports, due to its association with performance (5, 6). Adiposity, acting as a burden, leads to most sports requiring low levels of body fat to enhance movements and achieve greater efficiency, and consequently, better outcomes (7, 8).

Different methods have been employed to accurately estimate fat mass, all of them with advantages and disadvantages (9–11). Dual-energy X-ray absorptiometry (DXA) is considered by the scientific community as the criterion method, but the high-cost leaves DXA body composition assessments for very high-resource institutions (12). On the other hand, anthropometry and bioimpedance (BIA) are the most popular methods due to their low-moderate cost (12). Also, their “portability” has made them the most widely used in both sports and clinical practice (13).

In relation to anthropometry, it stands out as a low-cost method compared to other methods. In addition, it is transportable, which is very useful for measuring athletes and in the clinical field (1, 8, 12). Furthermore, it is a method that allows replicability by other trained researchers who follow a standardised method, such as the ISAK protocol (14, 15).

In relation to BIA, it is important to mention that it can be conducted using four different technologies (e.g., leg-to-leg, hand-to-hand, foot-to-hand, and standing position) (16) and with the subject in different measurement positions (i.e., standing, supine, or sitting). This is relevant given that the fat mass results reported by the BIA may depend on these factors, although these aspects in relation to the technology and position are often not taken into account to assess their accuracy and validity (17). While the supine position is the most commonly used position in research (18), the standing position is more commonly used in the clinical field, being the position chosen for assessment by the majority of BIA devices (19). However, standing BIA in most cases does not provide electrical conductivity data, i.e., raw bioimpedance parameters (20). Therefore, the equations that applies this type of device to estimate fat mass and the reference values it uses are exclusively those included in the software of the specific BIA model being used (16, 21).

It is important to note that while all these methods of fat mass estimation call the estimated adiposity as “fat mass” in the different reports and software, they are taking different approaches to estimate adiposity and, therefore, measuring different things (12, 22). More specifically, regardless of the method used, body composition can be approached based on five levels of increasing complexity (23). Most popular are model 2, which takes a molecular approach to body composition, considers body mass as the sum of fat mass and fat-free mass, including water, proteins, carbohydrates, and minerals; and model 4, which takes a tissue approach to body composition, considers body mass as the sum of adipose tissue, skeletal muscle tissue, bone tissue, skin tissue, and residual tissue (23). Accordingly, depending on the approximation model used by the technique chosen for the estimation of body composition, fat mass values are obtained if a molecular approach is used (model 2); or adipose tissue, if a tissue approach is used (model 4) (8, 22). And although both terms are classically lumped together under the heading “fat mass” (22), adipose tissue encompasses all components of the adipocyte (adipose cell), while fat mass corresponds to the lipid fraction of the adipose cell (22). This leads to errors of approximately 8–10% in the estimation of “fat mass” when approaching adiposity from different models (8).

Regarding the model used by the different methods to address body composition, DXA approaches body composition from a molecular approach (model 2), using X-rays to distinguish materials based on their atomic number (24). These data are then transformed into pixels of varying colors representing lipids, bone minerals, and other molecules, thereby enabling the acquisition of fat mass values (24). BIA, also approach from model 2, and uses a molecular fractionation to body composition (model 2) analysing molecular conductance and resistance to the flow of an electric current, estimating fat mass (9, 25). In anthropometry, through the measurement of skinfold thickness, in most cases seeks to estimate body density for subsequent fat mass estimation, according to model 2 (molecular model) (23, 26), dividing between fat mass and fat-free mass as the previous methods mentioned (27–30). However, anthropometry also seeks to approximate body composition according to model 4 (tissue model). This is done only through the Kerr formula, which is a mathematical model for estimating adipose tissue, which was validated in tissue fractionation of cadavers (30–33). As a consequence of the above, the results of the Kerr equation cannot be compared with those obtained for the other formulas (8) and it is necessary to convert the adipose tissue result of the Kerr equation to fat mass if you want to compare the results.

As a result of the above, different studies that have compared the results reported for fat mass by DXA, BIA, and anthropometry (34–36); DXA and BIA (37, 38); BIA and anthropometry (39, 40); or different anthropometry formulas (8, 41), have reported that there are differences between all of them in the results reported, making the results not comparable. However, only two of these studies took into account the need to fit the results reported by the different systems to the same model in order to be able to compare them. More specifically, an investigation of 32 young people (19 males and 13 females) compared the results reported by DXA; air displacement plethysmography, underwater weighing (UWW) and BIA. After fitting all results to obtain comparable results, it was found that the differences between the results reported by most of these methods disappeared after fitting to the same model (38). In another study of 87 subjects, different anthropometric formulas were compared for the estimation of “fat mass,” adjusting adipose tissue to fat mass, and it was found that even after adjustment there were significant differences between the results reported by the different formulas (8). Therefore, so far, no research has addressed this issue by including the three most popular methods currently used to estimate adiposity, i.e., DXA, anthropometry and BIA.

In view of the above, the objectives of this research were to: (a) determine the agreement of DXA with both BIA and anthropometry for estimating body fat mass and to analyse if sex and hydration status affect these results; and (b) analyse the validity of both anthropometry and BIA versus DXA for fat mass estimation, as well as the different formulas available, and whether sex and hydration status could affect this issue. Considering the findings of previous studies, the research hypothesis were: (a) anthropometry and BIA are not interchangeable to estimate fat mass respect to DXA; and (b) not all methods of assessing body composition are valid in estimating fat mass compared to DXA.

## 2 Materials and methods

### 2.1 Design

The present research followed a descriptive, cross-sectional design. The sample recruitment was non-probabilistic by convenience. The calculation used to establish the minimum sample size was performed with Rstudio 3.15.0 software (Rstudio Inc., Boston, MA, United States). The significance level was set at *α* = 0.05. The standard deviation (SD) for the total sample was set based on previous studies on the variables of fat mass percentage (SD = 5.19) (8). This methodology for sample size calculation has been used in previous research (42). Thus, the minimum sample size was 265 subjects, assuming an error (*d*) of 0.62% for fat mass percentage and for a 99% confidence interval (CI). Considering that acceptable statistical power is greater than 0.80 (43). The calculated statistical power was 0.96, which is high.

The Ethics Committee from the Catholic University San Antonio of Murcia (Murcia, Spain) reviewed and authorized the protocol designed for data collection, considering the World Medical Association Code (CE062103). All recommendations from the Declaration of Helsinki were followed throughout the process. Participants were informed about the procedure and signed a consent form prior to starting the study.

### 2.2 Participants

**Figure 1:**
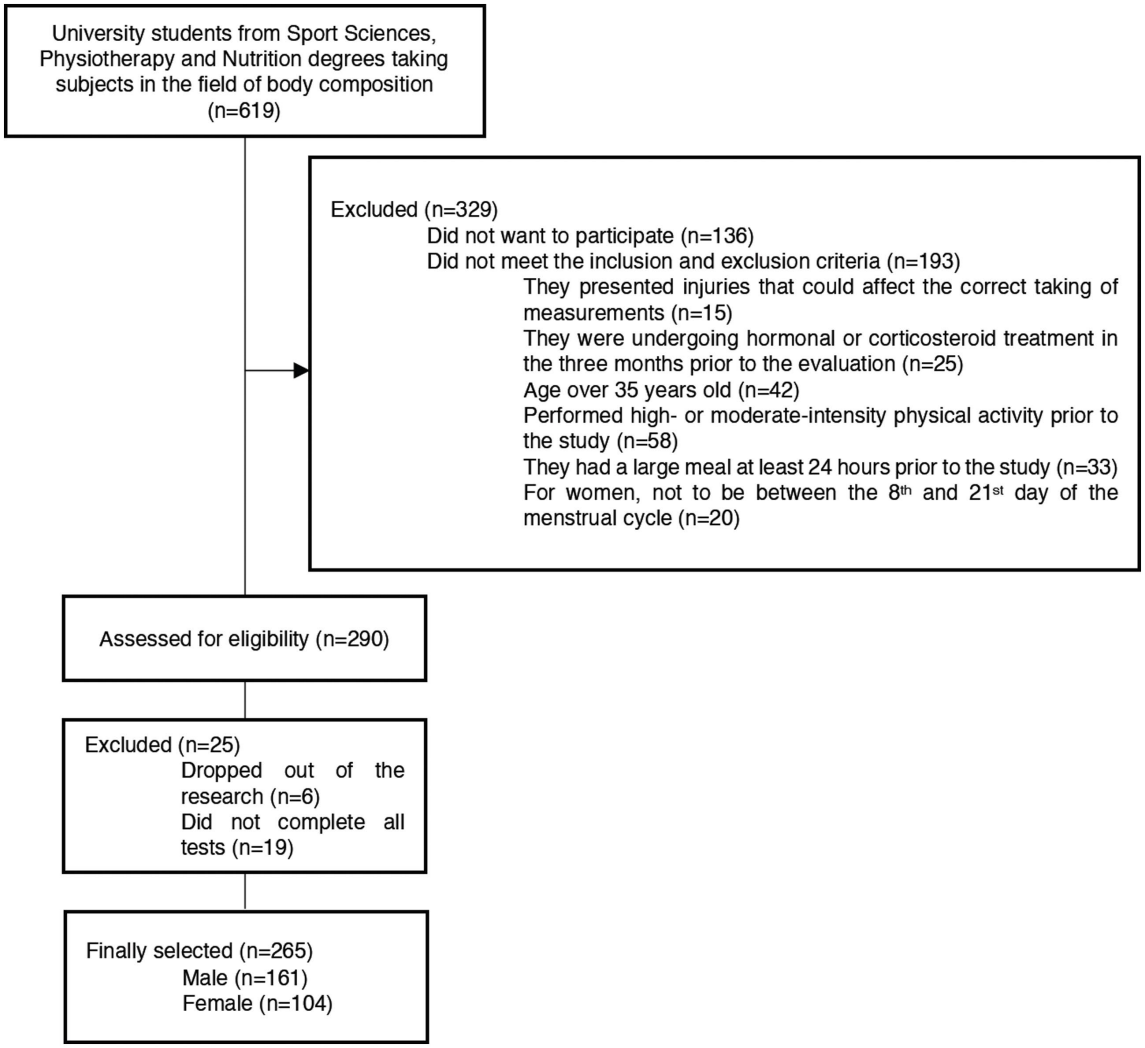
Participants’ flow chart.

A total of 265 volunteers were included, with their selection being non-probabilistic by convenience. Of these, 161 were male (mean age = 23.04 ± 5.61 years old); and 104 were female (mean age = 22.29 ± 5.98 years old). The flow diagram of the sample selection process can be consulted in Figure 1. The inclusion criteria were: (1) to be aged between 18 and 35 years old and (2) not having ingested any liquids and/or food from the night before the measurements. The exclusion criteria were: (1) to have performed vigorous physical exercise within the 24 h prior to the measurement session, or 12 h prior to the measurement in case of moderate exercise, or any kind of physical exercise on the same day of the measurement; (2) to have consumed products with diuretic properties, or eaten a heavy meal within the 24 h prior to the measurement session; (3) to have any injury or pathology that conditioned the taking of measurements; (4) to have any disease that could affect body fat; (5) to take hormonal or corticosteroid treatment in the three months prior to the evaluation (except for hormonal treatment to regulate the menstrual cycle); (6) for women, not to be between the 8th and 21st day of the menstrual cycle; (7) to take sports supplements that could impact fat distribution or the validity of body composition estimation methods, such as creatine or fat burners; and (8) failure to complete all measurements.

### 2.3 Protocol

Within the university subjects related to body composition in the degree of Nutrition, Physiotherapy, and Sport Sciences at the UCAM Universidad Católica San Antonio de Murcia (Murcia, Spain), an announcement was posted in the virtual classroom to invite students to volunteer for the study. They were required to fill out an initial questionnaire with basic data, and those who met the inclusion criteria were contacted to provide further instructions. Informed consent was obtained from each participant, and appointments for the measurements were scheduled, considering the menstrual cycle in the case of females. All appointments and measurements were carried out in the morning between 8 am and 10 am. All persons included were fasting from the previous night.

Firstly, a urine sample was taken from all participants to assess their hydration levels. The sample was used to classify the hydration status according to the colour of their urine and according to their urine density, measured with a refractometer (44, 45). Then, the participants were asked to complete an *ad hoc* questionnaire to provide information on basic sociodemographic information (sex, date of birth, and ethnicity), diseases that could affect body fat accumulation or distribution (illnesses and injuries in the last six months, chronic illness and surgeries), medication taken regularly, hormonal or corticosteroid treatment (daily or occasional treatment in the last six months), last menstrual period for women, food intake (24 h dietary recall), and sports practice (48 h exercise recall), based on previous studies (46, 47). They were also asked about sports supplements taken regularly.

Secondly, all the body composition measurements with DXA, anthropometry, and BIA were performed in a single session day in a room with a standardized temperature of 24°C. The tests were performed in a randomised order, with the measurements in each test taken by the same individual in all measurement sessions to eliminate inter-rater technical error in the test.

#### 2.3.1 Dual-energy X-ray absorptiometry

A total body composition measurement by DXA was performed on each participant. The Hologic Horizon model (Hologic Inc., Bedford, MA, United States) was used. The assessment was carried out by the same expert technician with previous experience. For the assessments, all participants were provided with sports tights and previous protocols were followed, including the removal of all metallic elements that could alter the results, and all participants were also asked to urinate within 30 min before the measurements (48). In the scanner, subjects were positioned with their hands in a lateral position and both feet in a 15° internal position (48). Results were analysed using Hologic APEX 13.6.0.5:5 software (Hologic Inc., Bedford, MA, United States). The values for fat mass (kg and percentage) were measured (Table 1).

**Table 1:** Methods and equations for the estimation of fat mass included in the present study.

	Fat mass
Dual-energy X-ray absorptiometry (DXA)	Value report by Hologic Horizon software
Bioimpedance (BIA)	Value report by TANITA MC-780-MA software
Anthropometry	Fat mass derived from the Kerr formula after Martin conversion
	Fat mass derived from the Kerr formula after Snyder conversion
	CUNBAE
	Forsyth et al.
	Evans et al.
	Carter et al.
	Brozek et al.
	Yuhasz et al.
	Faulkner et al.
	Durnin-Womersley et al.

#### 2.3.2 Bioimpedance analysis

Each subject was measured by BIA with the TANITA MC-780-MA model (Tanita Corporation, Tokyo, Japan), which operates by segmental multifrequency (measuring frequencies: 5 kHz/50 kHz/250 kHz) and consists of eight electrodes. The subjects were measured in a standing position, following the technical instructions in the user manual of the device. All protocols established by the manufacturer were followed, including urinating within 30 min before the measurements. Water consumption or dietary factors that may affect total body water levels were also asked about (49). All subjects were measured in sports tights, and all metallic elements were removed from their bodies. Body mass values were analyzed, as well as percentage and kilograms of fat mass with the TANITA software (Tanita Corporation, Tokyo, Japan) (Table 1).

#### 2.3.3 Anthropometry

The anthropometric variables were taken following the protocol by the International Society for the Advancement of Kinanthropometry (ISAK). Body mass and height; triceps, subscapular, biceps, iliac crest, supraspinal, abdominal, thigh, and calf skinfolds, were taken by the same ISAK level-3 certified anthropometrist. The skinfold measurements were taken on the right side according to the ISAK protocol (14) at the third second after the full pressure of the calliper was applied (26). A TANITA MC-780-MA model (Tanita Corporation, Tokyo, Japan), with an accuracy of 0.1 kg, was used to measure body mass; a portable stadiometer (SECA, Hamburg, Germany), with an accuracy of 0.1 cm, was used for height measurements, and a Harpenden calliper (Harpenden, London, United Kingdom), with an accuracy of 0.2 mm, was used for skinfolds. Each measurement was taken twice. If the difference between them was greater than 1% for the basic measurements or greater than 5% for the skinfolds, a third measurement was taken. The final value considered for data analysis was the mean if two measurements were taken, or the median if three measurements were taken (50). The intra-evaluator technical error of measurement (TEM) was 0.01% for basic measurements and 1.12% for skinfolds (50). For the estimation of fat mass and its percentages, formulas have been selected because they have been used in previous studies with similar samples (8). In addition, these are the validated formulas most commonly used in the young population (8). They were proposed by CUNBAE (51), Forsyth and Sinning (52), Evans et al. (29), Carter (27), Brozek and Keys (53), Yuhasz (54), Faulkner (28), and Durnin and Womersley (30). The formula proposed by Kerr and Ross (33) was also included, although as it is a formula that estimates adipose tissue, its transformation into fat mass was calculated by a linear regression assuming a minimum percentage error with Martin’s et al. (55) formula and Snyder’s et al. (56) formula (Table 1).

### 2.4 Statistical analysis

The normal distribution with Kolmogorov–Smirnov test, kurtosis and asymmetry of the variables were calculated. Levene’s test was used to assess the homogeneity of the variables. Levene’s test was used to assess the homogeneity of the variables. The analysis of skewness and kurtosis showed a platykurtic distribution for all variables. As a normal and homogeneous distribution of the variables was found, parametric tests were performed. Descriptive statistics were performed for the variables analyzed. Differences between the fat mass equations and methods were analysed with an ANOVA test for repeated measurements. An ANCOVA for repeated measurements was used to analyse the influence of the variables “sex” and “hydration status” on the differences found. The Bonferroni *post hoc* adjustment was used to analyse differences between DXA and anthropometry and BIA formulas when these differences were significant. The effect size for the pairwise comparisons was calculated with partial Eta-squared (*η*^2^*_p_*). The confidence interval (CI) of the differences (95% CI) was included. The software used in the statistical analysis was SPSS (v.23, IBM, Endicott, NY, United States). The agreement between equations and methods was determined using Lin’s concordance correlation coefficient (CCC), including precision (*ρ*) and accuracy (Cb) indexes, as well as with McBride’s strength concordance (almost perfect >0.99; substantial >0.95 to 0.99; moderate = 0.90–0.95; and poor <0.90), following previous research (46). The Bland–Altman test was used to determine the validity of different anthropometry and BIA equations with respect to DXA values. The trend to overestimate or underestimate the values respect to the reference method and the regression equation for the model was also calculated. The software used to perform the Lin’s CCC and Bland–Altman test was MedCalc Statistical Software v.20.106 (Mariakerke, Belgium). For all the statistical tests, the significance level was set *a priori* to *p* ≤ 0.05.

## 3 Results

Table 2 and Supplementary Figures S1–S6 shows the results of the descriptive statistical analysis for the general population, male and female samples, including means, standard deviation, a minimum and maximum values.

**Table 2:** Descriptive analysis of fat mass in kilograms and percentages in the general sample and separated into male and female samples.

Method and formulas	General sample (*n* = 265)		Male sample (*n* = 161)		Female sample (*n* = 104)	
	Mean ± SD	Min.–Max.	Mean ± SD	Min.–Max.	Mean ± SD	Min.–Max.
DXA in kg	12.50 ± 6.43	0.18; 40.57	15.69 ± 5.66	3.16; 38.67	15.31 ± 6.56	0.18; 40.57
BIA in kg	14.72 ± 6.06	1.90; 42.10	13.89 ± 5.75	1.90; 40.00	15.99 ± 6.32	5.00; 42.10
ANTHR Kerr converted by Martin in kg	16.40 ± 6.71	6.46; 41.17	14.71 ± 5.97	6.46; 37.12	19.02 ± 6.99	7.26; 41.17
ANTHR Kerr converted by Snyder in kg	18.21 ± 5.28	9.13; 36.82	17.62 ± 5.15	9.78; 36.82	19.13 ± 5.38	9.13; 36.35
ANTHR CUNBAE in kg	19.88 ± 10.02	1.70; 49.14	25.82 ± 7.43	12.32; 49.14	10.69 ± 5.59	1.70; 37.43
ANTHR Forsyth in kg	13.40 ± 8.70	2.98; 57.63	14.17 ± 8.73	3.84; 47.49	12.19 ± 8.55	2.98; 57.63
ANTHR Evans in kg	14.28 ± 5.92	4.45; 38.26	14.08 ± 5.94	4.45; 38.26	14.60 ± 5.91	5.27; 34.99
ANTHR Carter in kg	12.76 ± 5.85	4.28; 40.39	12.38 ± 5.66	5.29; 36.49	13.35 ± 6.13	4.28; 40.39
ANTHR Brozek in kg	18.15 ± 6.08	7.14; 41.14	18.66 ± 6.18	8.77; 40.21	17.36 ± 5.86	7.14; 41.14
ANTHR Yuhasz in kg	10.57 ± 5.36	3.70; 41.52	8.77 ± 3.92	3.70; 24.96	13.36 ± 6.08	4.61; 41.52
ANTHR Faulkner in kg	14.45 ± 5.84	5.70; 40.41	14.97 ± 5.79	7.34; 37.54	13.63 ± 5.86	5.70; 40.41
ANTHR Durnin in kg	18.53 ± 6.43	7.01; 42.94	18.98 ± 6.56	8.59; 41.80	17.84 ± 6.19	7.01; 42.94
DXA in %	17.68 ± 8.24	0.44; 43.15	13.38 ± 5.77	5.08; 34.68	24.35 ± 6.97	0.44; 43.15
BIA in %	20.55 ± 6.92	3.00; 41.00	17.34 ± 5.21	3.00; 35.90	25.51 ± 6.28	9.80; 41.00
ANTHR Kerr converted by Martin in %	23.44 ± 9.26	8.54; 49.40	18.69 ± 6.37	8.54; 41.30	30.79 ± 8.20	14.95; 49.40
ANTHR Kerr converted by Snyder in %	25.84 ± 6.63	13.27; 42.06	22.44 ± 4.98	13.27; 37.73	31.12 ± 5.30	19.64; 42.06
ANTHR CUNBAE in %	26.27 ± 9.31	3.95; 45.03	32.45 ± 4.95	20.61; 45.03	16.70 ± 5.65	3.95; 36.41
ANTHR Forsyth in %	18.08 ± 8.98	5.96; 56.06	17.53 ± 8.71	5.96; 46.65	18.92 ± 9.37	6.08; 56.06
ANTHR Evans in %	19.95 ± 6.51	6.85; 40.84	17.72 ± 5.84	6.85; 34.92	23.39 ± 6.00	10.37; 40.84
ANTHR Carter in %	17.70 ± 6.18	8.13; 40.43	15.47 ± 5.19	8.13; 32.72	21.16 ± 6.02	10.07; 40.43
ANTHR Brozek in %	25.23 ± 5.63	13.71; 40.02	23.52 ± 5.31	13.71; 36.06	27.88 ± 5.08	15.59; 40.02
ANTHR Yuhasz in %	14.99 ± 6.84	5.74; 40.57	10.98 ± 3.62	5.74; 22.96	21.21 ± 5.94	10.83; 40.57
ANTHR Faulkner in %	19.92 ± 5.36	12.26; 39.31	18.79 ± 4.95	12.26; 34.92	21.68 ± 5.52	12.96; 39.31
ANTHR Durnin in %	25.75 ± 6.10	13.28; 41.77	23.90 ± 5.76	13.28; 37.49	28.62 ± 5.50	15.31; 41.77

DXA, dual-energy X-ray absorptiometry; BIA, bioimpedance analysis; ANTHR, anthropometry.

Table 3 presents the results of the ANOVA and the ANCOVA performed to analyze the effect of the covariates sex and hydration status in both the general sample and the sample separated according to sex. Significant differences were found between methods and equations used to estimate both kilograms and percentage of fat mass, in both the general sample (*p* < 0.001), and when the covariable sex was included (*p* < 0.001). In contrast, hydration status showed no significant effect on the model (*p* = 0.332–0.527). When the sample was divided according to sex, significant differences remained in both males and females for the fat mass estimation methods and equations, both in kilograms and in percentages (*p* < 0.001).

**Table 3:** Analysis of differences in fat mass (kg and percentage) between DXA, BIA, and anthropometry for the general sample and according to sex.

	ANOVA			Variable × Hydration status			Variable × Sex		
	*F*	*p*-value	*η* ^2^ *_p_*	*F*	*p*-value	*η* ^2^ *_p_*	*F*	*p*-value	*η* ^2^ *_p_*
**General sample (*n* = 265)**									
Fat mass in kg	10.363	<0.001	0.038	0.401	0.527	0.002	103.100	<0.001	0.282
Fat mass in percentage	13.188	<0.001	0.048	0.946	0.332	0.004	112.854	<0.001	0.301
**Males (*n* = 161)**									
Fat mass in kg	52.953	<0.001	0.249						
Fat mass in percentage	53.564	<0.001	0.251						
**Females (*n* = 104)**									
Fat mass in kg	49.519	<0.001	0.325						
Fat mass in percentage	51.427	<0.001	0.333						

When comparing the results shown by DXA with anthropometry and BIA in the general sample, the Bonferroni adjustment showed that DXA has significant differences with respect to all the other methods in kg (*p* < 0.001; 95% CI: −9.633, 2.429) and percentage (*p* < 0.001; 95% CI: −11.547, −7.294), with the exception of Carter’s formula in kg (*p* = 0.488; 95% CI: −2.027, 0.235) and percentage (*p* = 1.000; 95% CI: −0.863; 0.819), and Forsyth’s formula in kg (*p* = 1.000; 95% CI: −0.860, 0.335) and percentage (*p* = 1.000; 95% CI: −1.872, 1.086). When the sample was divided according to sex, significant differences remained between all the methods in male sample both for kg (*p* < 0.001; 95% CI: −16.586, 2.588) and percentage (*p* < 0.001; 95% CI: −20.494, −17.652). Regarding females, significant differences remained in females for the fat mass estimation methods in kilograms (*p* < 0.001; 95% CI: −4.907, 5.783) and percentage (*p* < 0.001; 95% CI: −8.452, 9.540), with the exception of BIA’s formula for kg (*p* = 0.127; 95% CI: −1.432, 0.062) and percentage (*p* = 0.109; 95% IC: −2.413, 0.086), and Evans’s formula for kilograms (*p* = 1.000; 95% CI: −0.337, 1.743) and percentage (*p* = 1.000; 95% IC: −0.743, 2.656).

**Figure 2:**
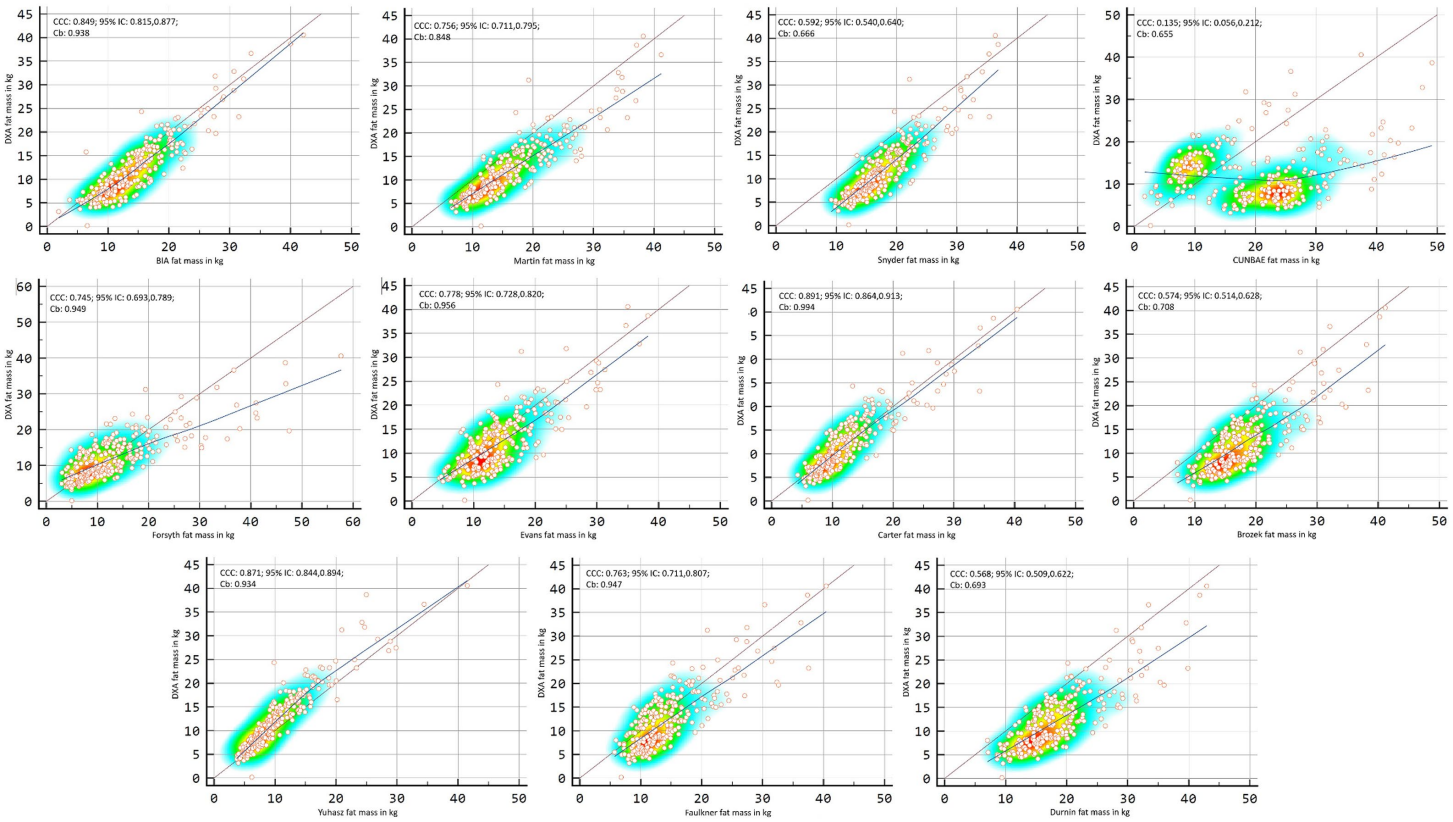
Point plot of the concordance between methods of fat mass in kg reported by DXA in the general sample.

**Figure 3:**
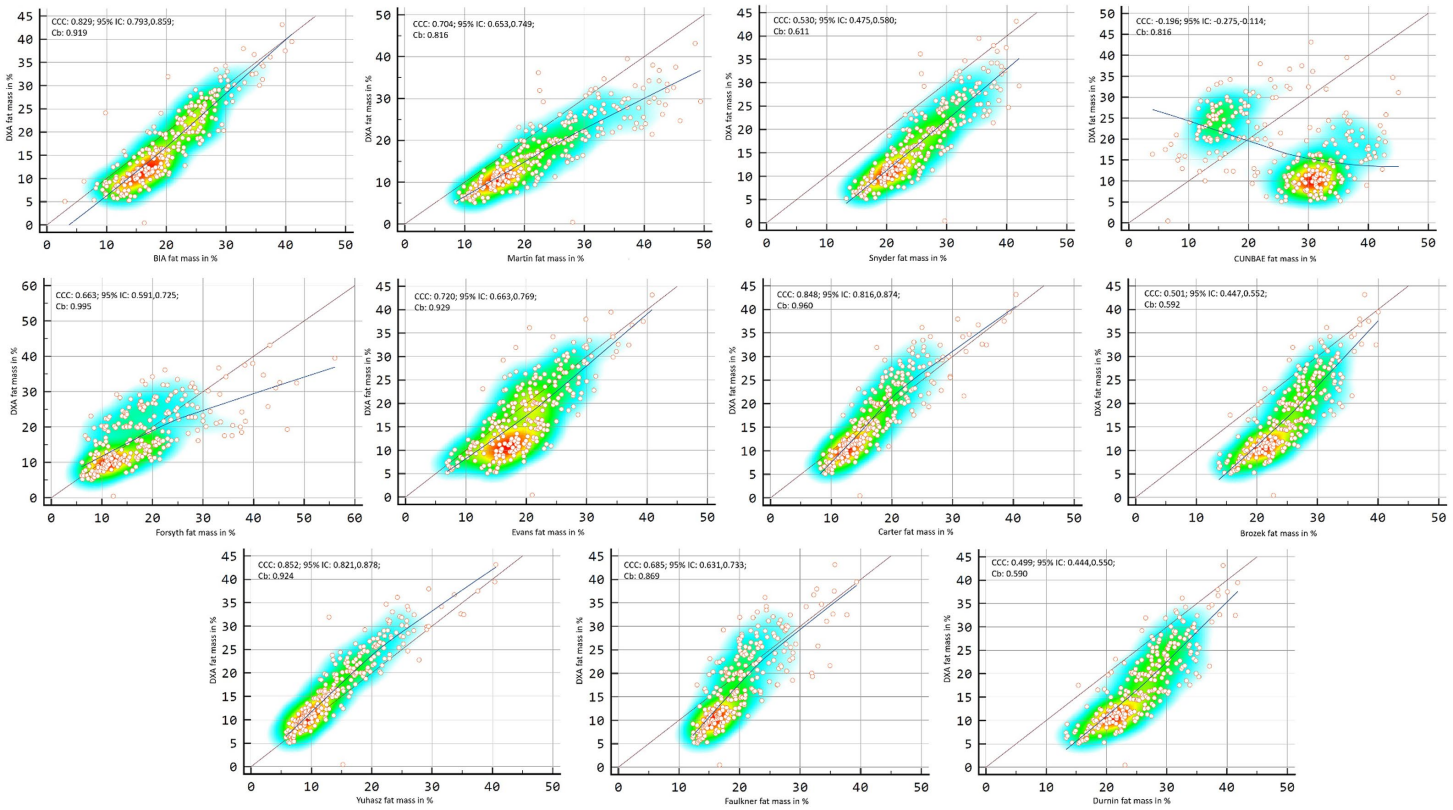
Point plot of the concordance between methods of fat mass in percentage reported by DXA in the general sample.

**Figure 4:**
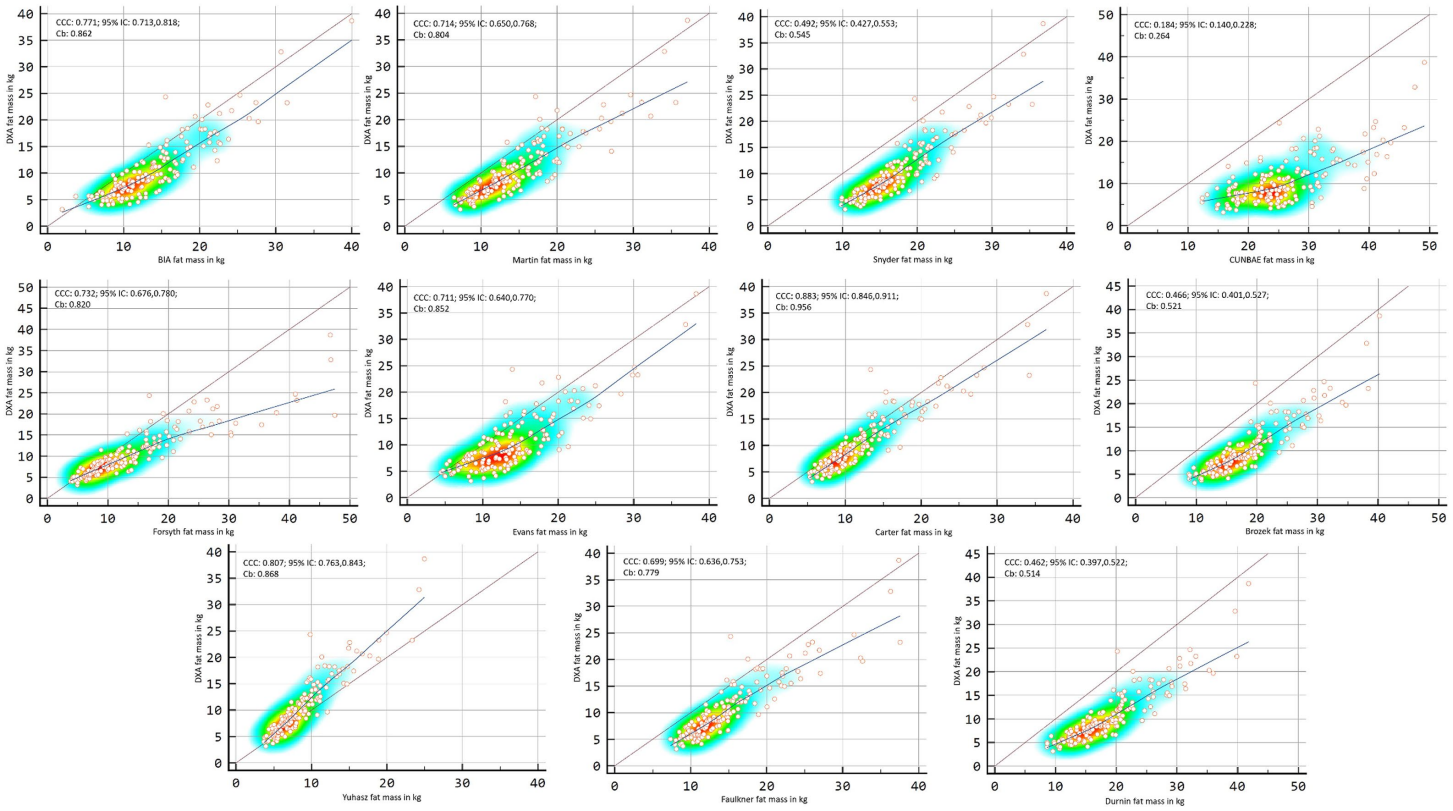
Point plot of the concordance between methods of fat mass in kg reported by DXA in males.

**Figure 5:**
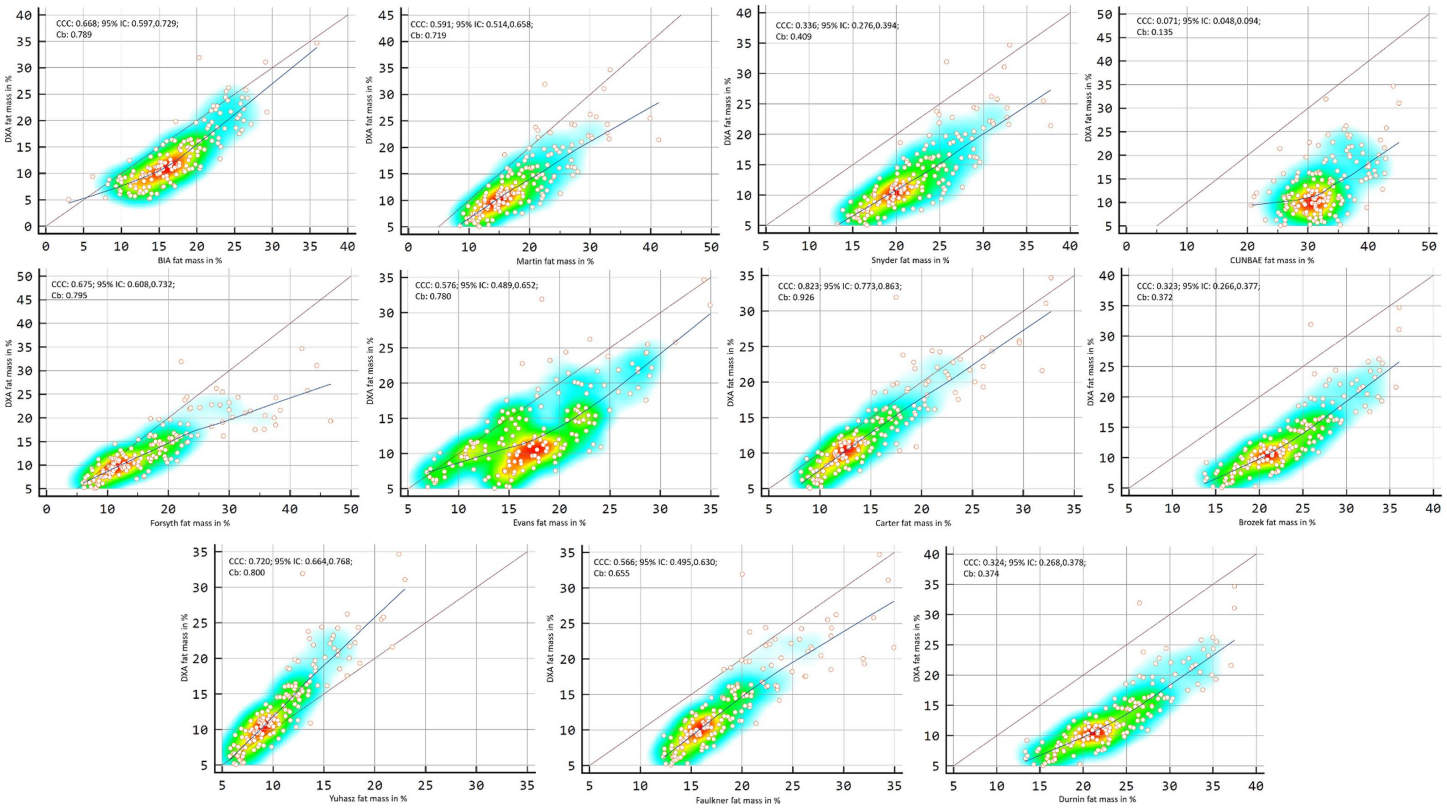
Point plot of the concordance between methods of fat mass in percentage reported by DXA in males.

**Figure 6:**
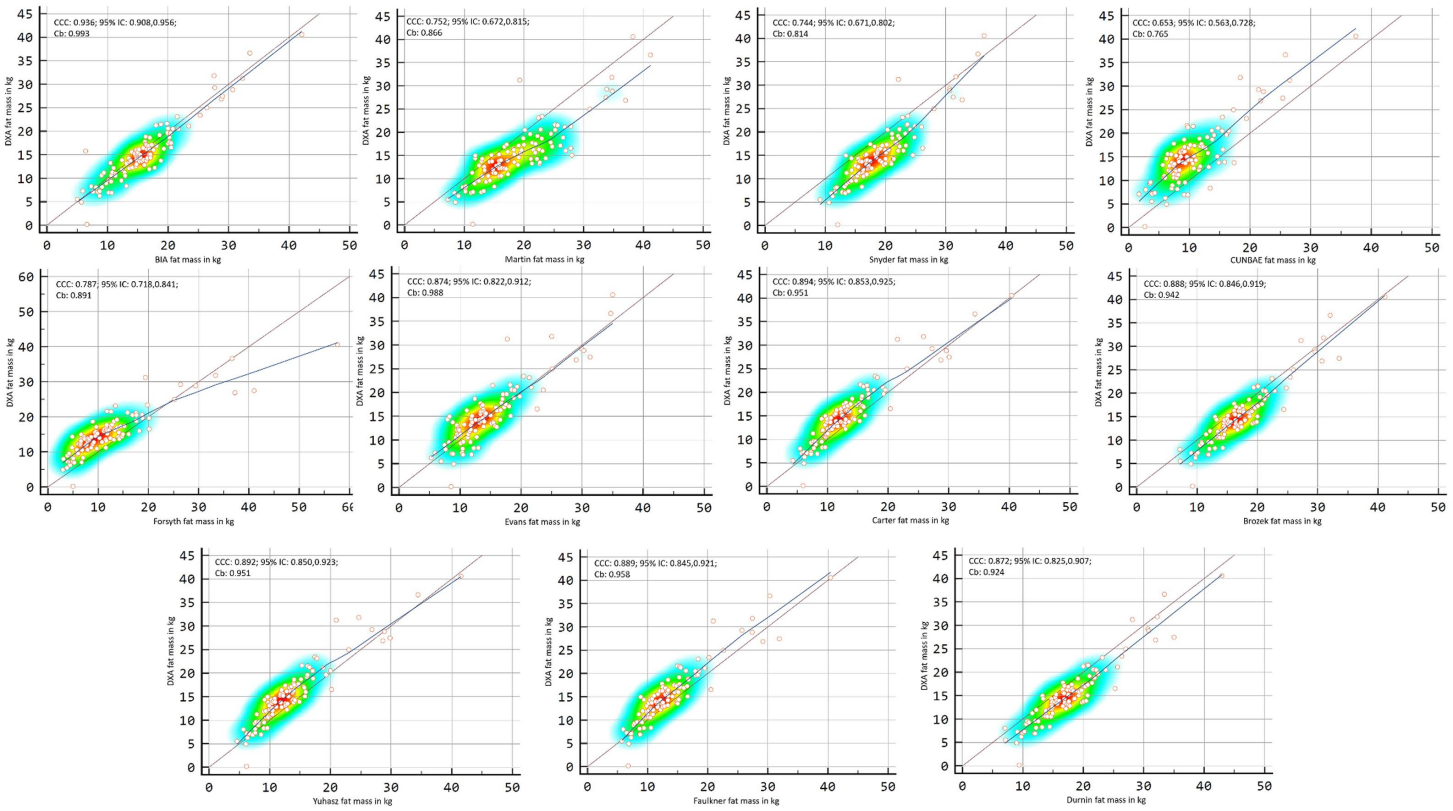
Point plot of the concordance between methods of fat mass in kg reported by DXA in females.

**Figure 7:**
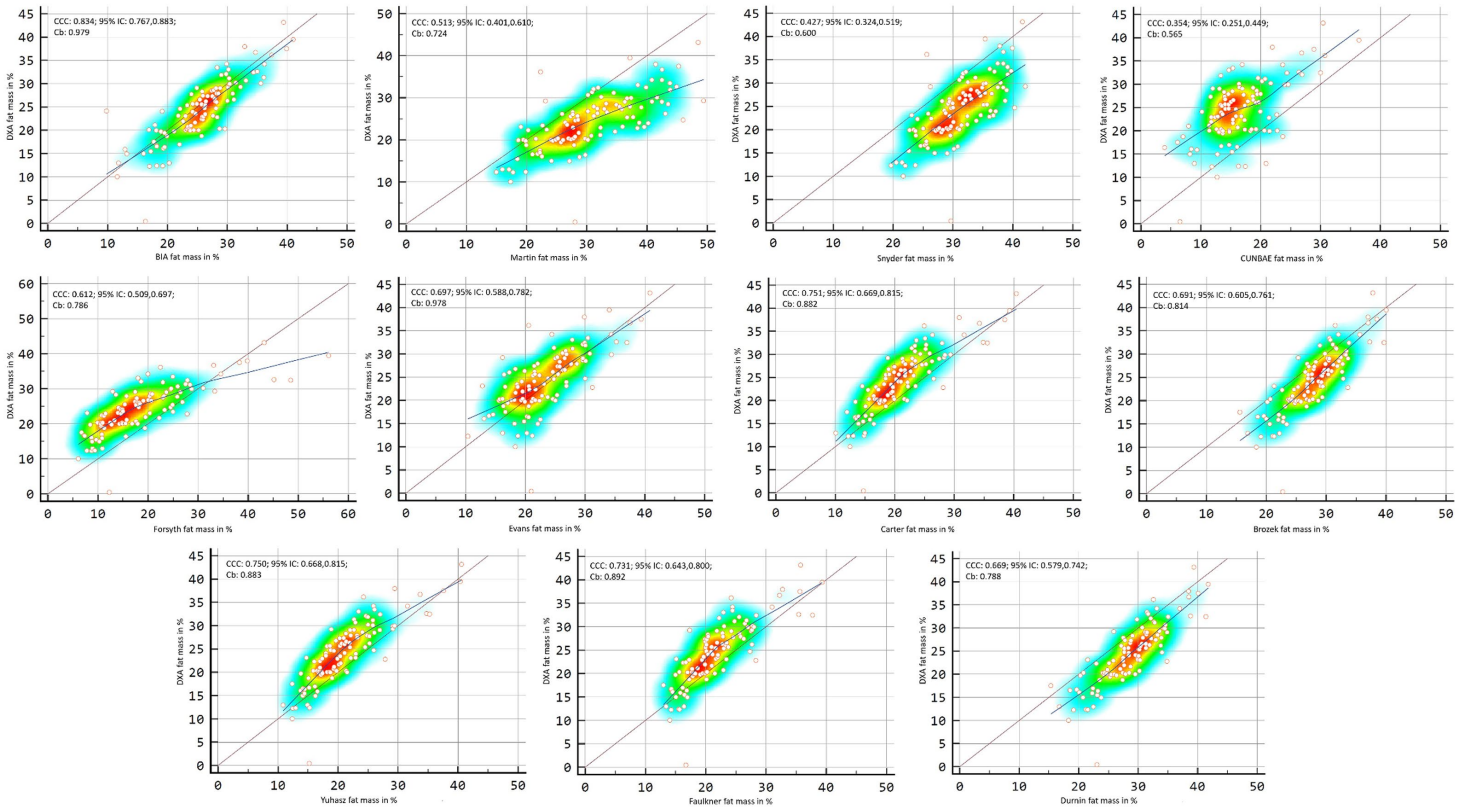
Point plot of the concordance between methods of fat mass in percentage reported by DXA in females.

Figures 2, 3 showed Lin’s coefficient for general sample in kg and percentage; Figures 4, 5 for males and Figures 6, 7 for females. Lin’s coefficient indicated than when the sample was treated as a group poor agreement between all the formulas and methods both in percentage and kilograms of fat mass (CCC = 0.135–0.892), with the exception of DXA’s and BIA’s formula of kilograms in female group (CCC = 0.936).

In the general group, the formulas that showed the statistically significant trend to overestimate fat mass compared with the DXA in kg and percentage were the Martin’s (*r* = 0.245, *p* < 0.001; and *r* = 0.321, *p* < 0.001), CUNBAE’s (*r* = 0.498, *p* < 0.001; and *r* = 0.137, *p* = 0.026), Forsyth’s (*r* = 0.593, *p* < 0.001; and *r* = 0.446, *p* < 0.001), Carter’s (*r* = 0.279, *p* < 0.001; and *r* = 0.235, *p* < 0.001) and Faulkner’s formulas (*r* = 0.145, *p* = 0.018; and *r* = −0.177, *p* = 0.004). The formulas that showed the statistically significant trend to underestimate fat mass and compared with the DXA were Evans’s formula (*r* = −0.798, *p* < 0.001) and Yuhasz’s formula (*r* = −0.898, *p* < 0.001) for kilograms; and BIA’s (*r* = −0.286, *p* < 0.001), Snyder’s (*r* = −0.368, *p* < 0.001), Brozek’s (*r* = −0.564, *p* < 0.001), Faulkner’s (*r* = −0.177, *p* = 0.004), and Durnin’s formulas (*r* = −0.481, *p* < 0.001) for percentage.

In males, the formulas that showed the statistically significant trend to overestimate fat mass compared with the DXA booth in kg and percentage were the Martin’s (*r* = 0.251, *p* < 0.001; and *r* = 0.296, *p* < 0.001) and Forsyth’s formulas (*r* = 0.756, *p* < 0.001; and *r* = 0.707, *p* < 0.001). The BIA’s (*r* = 0.155, *p* = 0.049), CUNBAE’s (*r* = 0.360, *p* < 0.001), Carter’s (*r* = 0.297, *p* < 0.001), Brozek’s (*r* = 0.238, *p* = 0.002), Faulkner’s (*r* = 0.173, *p* = 0.028) and Durnin’s (*r* = 0.361, *p* = <0.001) formulas showed trend to overestimate only in kilograms, and Evans’s (*r* = 0.162, *p* = 0.040) and Yuhasz’s (*r* = 0.128, *p* < 0.001) for percentage. The formulas that showed the statistically significant trend to underestimate fat mass and compared with the DXA were the Evans’s (*r* = −0.802, *p* < 0.001) and Yuhasz’s (*r* = −0.975, *p* < 0.001) for kilograms, and Snyder’s (*r* = −0.201, *p* = 0.010), CUNBAE’s (*r* = −0.179, *p* = 0.023), and Faulkner’s (*r* = −0.177, *p* = 0.024) for percentage.

In females, Martin’s formula showed statistically significant trend to overestimate fat mass compared with the DXA both in kg (*r* = 0.275, *p* = 0.005) and percentage (*r* = 0.256, *p* = 0.009). CUNBAE’s (*r* = 0.291, *p* = 0.003), Forsyth’s (*r* = 0.277, *p* = 0.004), Carter’s (*r* = 0.238, *p* = 0.015) and Faulkner’s (*r* = 0.342, *p* < 0.001) formulas showed trend to overestimate only in kilograms. The formulas that showed the statistically significant trend to underestimate fat mass compared with the DXA were Evans’s formula for kilograms (*r* = −0.833, *p* < 0.001) and percentage (*r* = −0.203, *p* = 0.039), Yuhasz’s formula for kilograms (*r* = −0.783, *p* < 0.001) and BIA’s (*r* = −0.246; *p* = 0.012), Snyder’s (*r* = −0.337, *p* < 0.001), Brozek’s (*r* = −0.452, *p* < 0.001), and Durnin’s (*r* = −0.384, *p* < 0.001) formulas for percentage.

**Figure 8:**
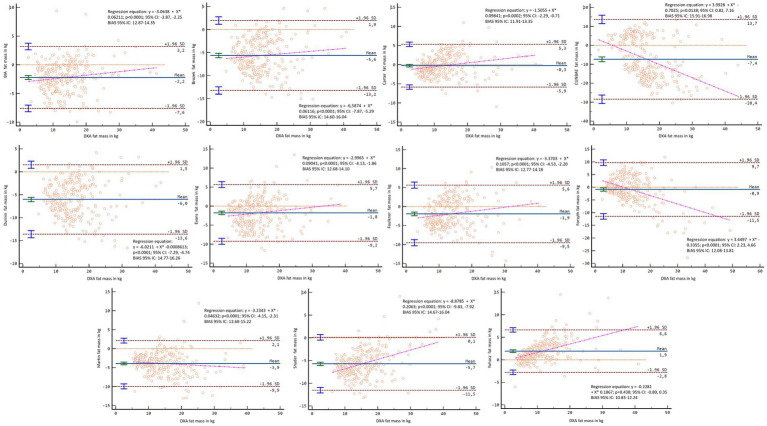
Bland–Altman analysis comparing all formulas and methods with the fat mass in kg reported by DXA in the general sample.

**Figure 9:**
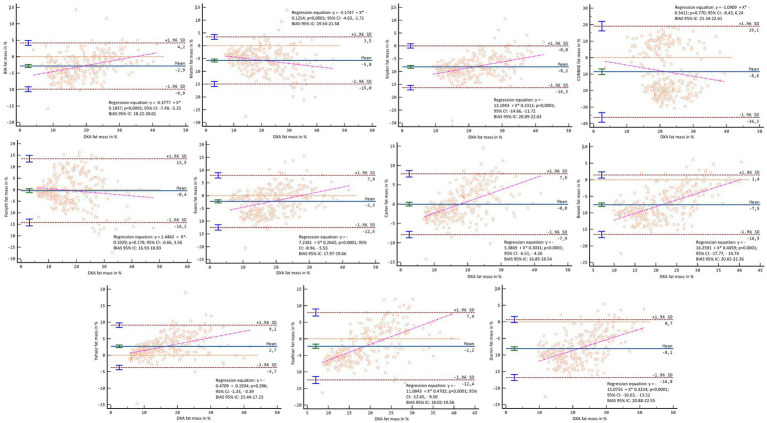
Bland–Altman analysis comparing all formulas and methods with the percentage of fat mass reported by DXA in the general sample.

**Figure 10:**
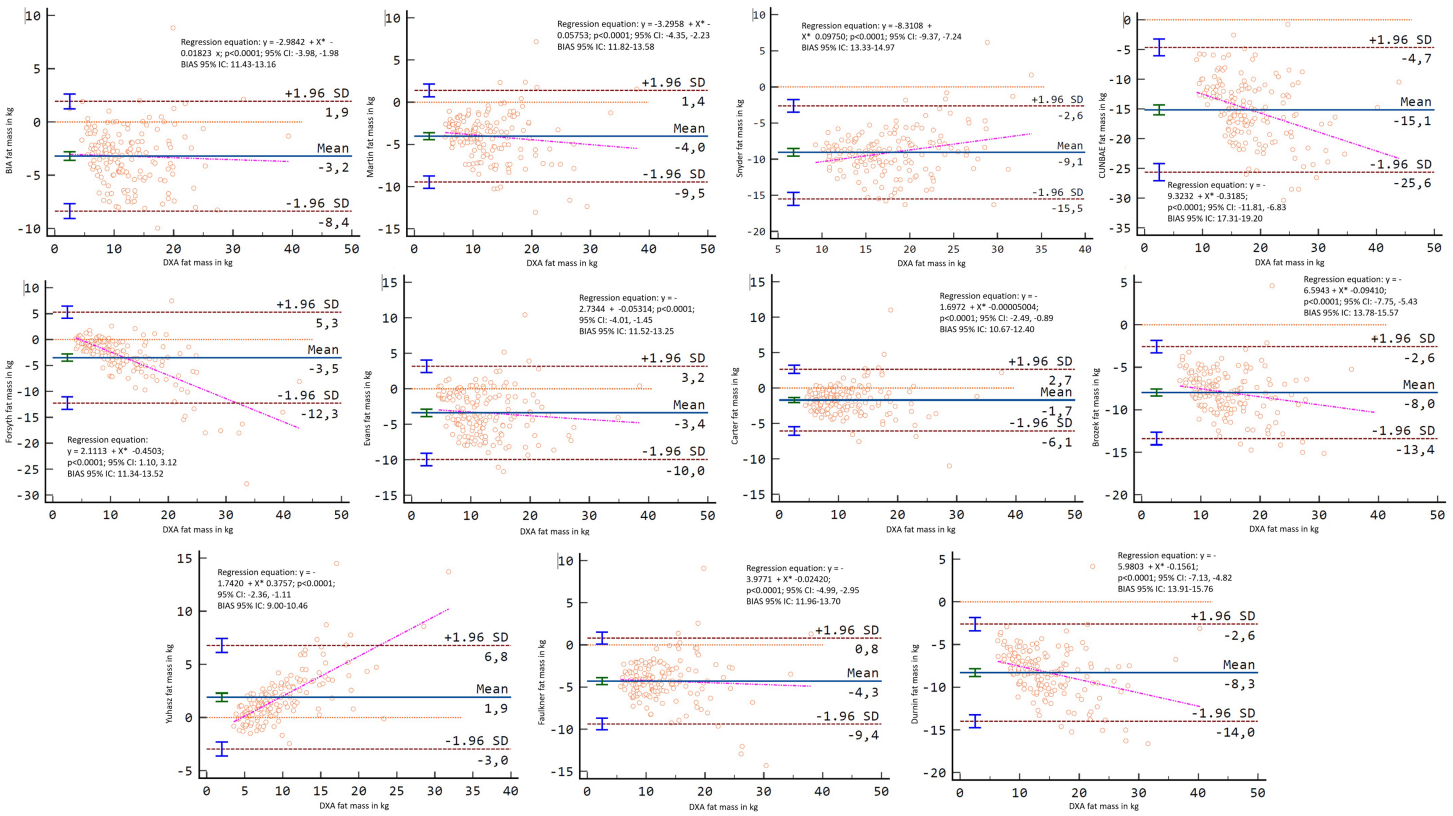
Bland–Altman analysis comparing all formulas and methods with the fat mass in kg reported by DXA in males.

**Figure 11:**
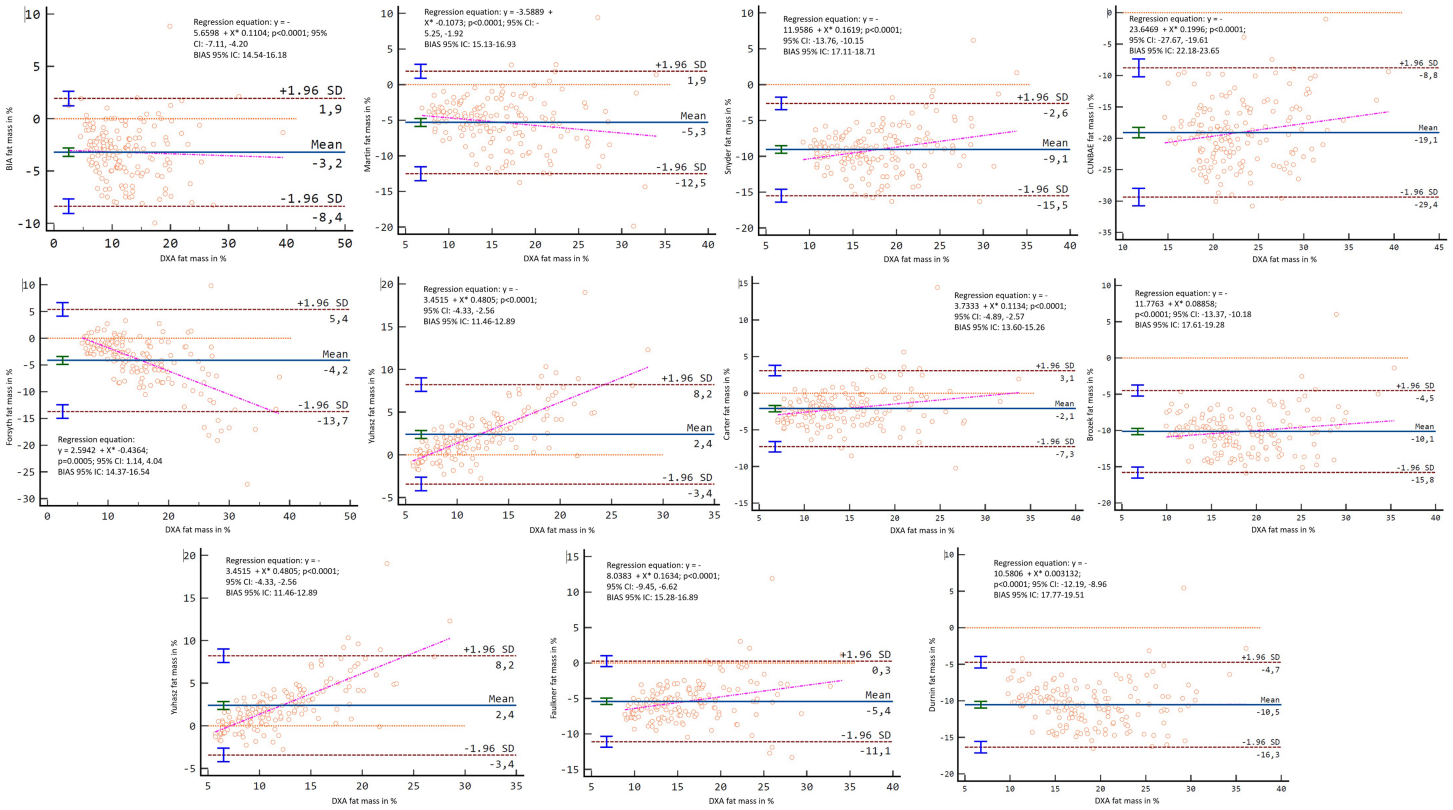
Bland–Altman analysis comparing all formulas and methods with the percentage of fat mass reported by DXA in males.

**Figure 12:**
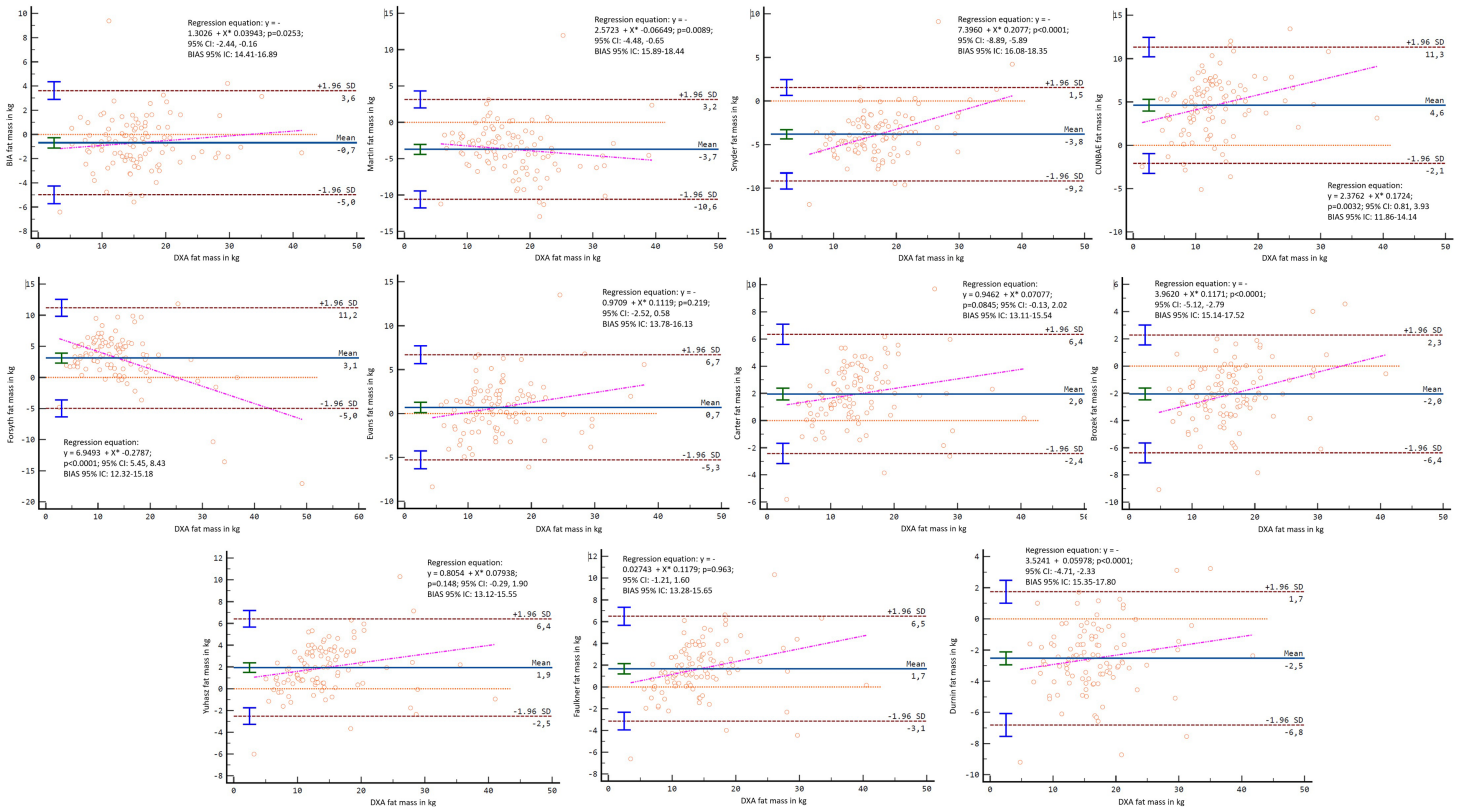
Bland–Altman analysis comparing all formulas and methods with the fat mass in kg reported by DXA in females.

**Figure 13:**
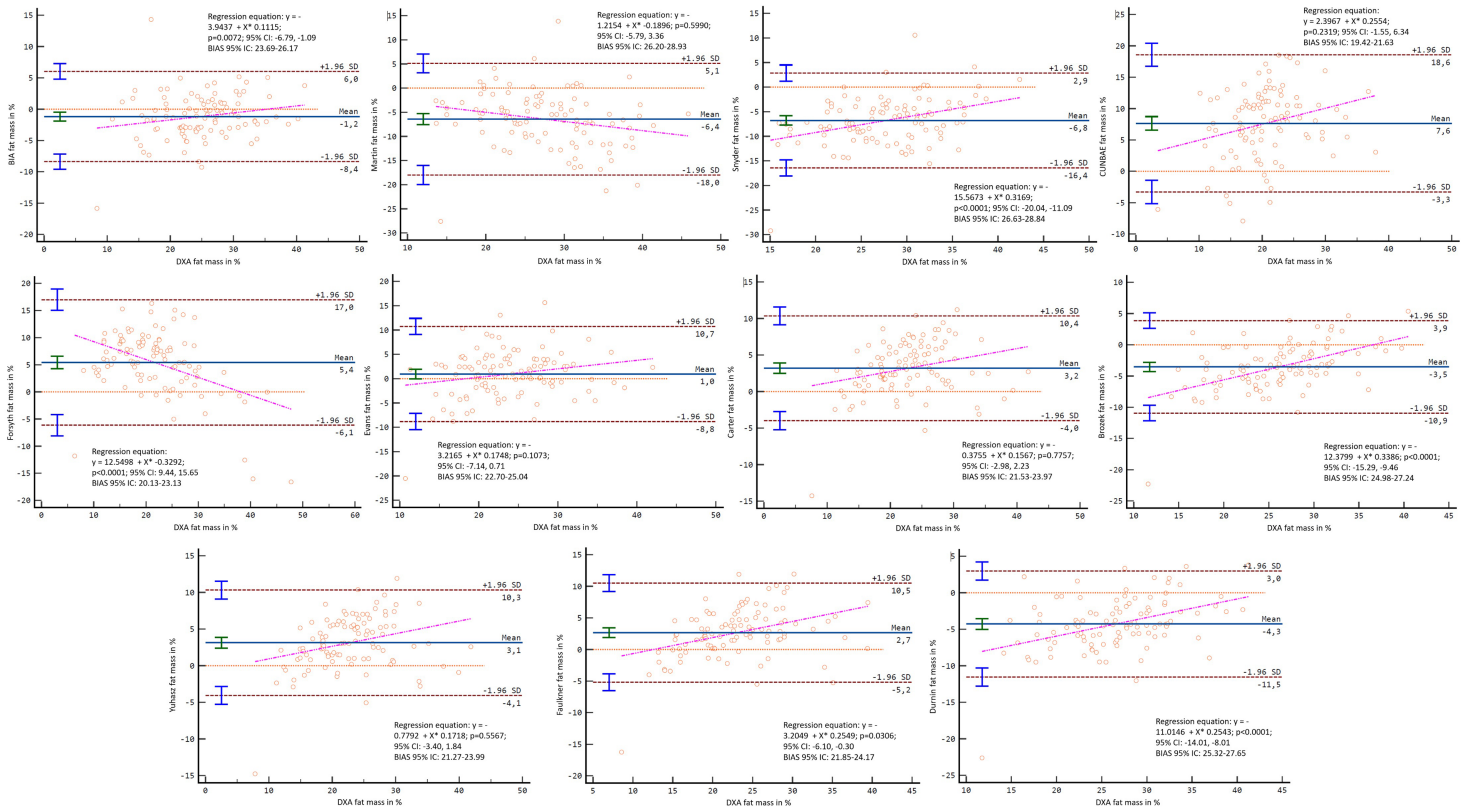
Bland–Altman analysis comparing all formulas and methods with the percentage of fat mass reported by DXA in females.

In the Bland–Altman analysis, using the DXA fat mass values as a reference, lack of agreement was found in the general sample (*p* < 0.001–0.007), except for Carter’s formula in kilograms (*p* = 0.136) and percentage (*p* = 0.929) and Forsyth for percentage (*p* = 0.365) (Figures 8, 9). When separating the sample by sex, lack of agreement was found in males for all methods when compared with both percentage and kilograms calculated by DXA (*p* < 0.001) (Figures 10, 11). In the female sample, all methods and formulas showed lack of agreement (*p* < 0.001–0.020), except for Evans’s in percentage (*p* = 0.058) (Figures 12, 13).

## 4 Discussion

The main objective of this research was to determine the agreement of DXA with both anthropometry and BIA for estimating fat mass; and to analyse the validity of both anthropometry and BIA versus DXA for fat mass estimation, as well as the different formulas available, and whether sex or hydration status could affect this issue. The main finding of the present investigation was that in general BIA and anthropometry showed significant differences respect fat mass value report by DXA, proving to be not very valid with respect to DXA; it was also found that there is no agreement for fat mass values in most cases neither when analysing populations nor when analysing individuals. Previous studies have also found differences when comparing DXA, BIA, and anthropometry (34–36) and DXA and BIA (37, 38). However, no previous study had previously compared the results found in the estimation of fat mass with the main methods used in the assessment of body composition (DXA, BIA, and anthropometry); adjusting the results according to the model used by each method; with as many formulas as included in this research, and with such a large sample.

The differences found in the results of fat mass from anthropometry vs. DXA may be a consequence of the fact that most of the formulas for estimating fat mass with anthropometry had been validated based on multiple regressions, and are specific to a population with similar characteristics to the one used in their validation, although for practical purposes, the lack of formulas validated in each of the different populations means that they are applied in different contexts (29, 30, 52). Thus, the application of these formulas and methods to people of other sex, other levels of physical activity, other races, other nutrition habits, other genetics, etc. is uncertain and unreliable in the light of the results of this research.

Another possible explanation could be that anthropometry has put forward different options for approaching the adipose component depending on the method used for its estimation. More specifically, while Kerr anthropometric formula, focus on a model 4 approach aiming to estimate subcutaneous adipose tissue as a whole, other anthropometric formulas focus on model 2 to estimate fat mass (8, 57). Although Martin’s and Snyder’s formulas for converting adipose tissue estimated by Kerr to fat mass was used in the present investigation to solve the problem of different models for approximating body composition (8, 22), no agreement was still found between Kerr’s fat mass and DXA results, so the appropriateness of Kerr’s method should be investigated in future research.

Regarding the reported differences in fat mass results between DXA and BIA, previous studies have also pointed out that BIA may be a method whose concordance and validity is limited with respect to DXA (58–60). This could be since previous studies, as in the present one, a BIA device was used that do not report the raw electrical conductivity data (61), but rather the device’s own software gives the fat mass estimation as a result (61, 62). Previous studies have suggested that the validity of BIA may be increased when the device gives the raw data and then this data is used by the researcher to estimate fat mass using the most appropriate formula for the population being analysed (21). A second justification for the lack of validity of the BIA results could be found in the fact that the device used in this research carried out the analysis in the standing position, which is the most common position in the clinical use of the BIA (63), but it has been shown that the validity of BIA analysis increases when the device is prepared to assess the subject in supine position (64), probably due to changes and stabilization in body water distribution that affects the results obtained (65). Therefore, future studies need to investigate these issues further.

Another outstanding result of the present research is that sex was an important factor to consider in the analysis of validity and agreement between methods. This is an interesting finding, considering that many of the formulas and methods for estimating fat mass have been classically used both in both men and women, regardless of the population in which they were validated (66). The different physiological characteristics between sex need to be considered. A relevant difference to the aim of the study is the tendency to accumulate adiposity (67, 68). In men, there is a greater accumulation in the central abdominal area of the body, while in women, this occurs more frequently in the gluteal-femoral area (67). So, in the light of the present research, it is important to consider sex when estimating body fat with these methods (69).

An important finding of the present research was that with respect to the agreement between formulas, the variable hydration status was not found to directly affect the results. Previous studies have shown a high impact of hydration levels on the results, mainly obtained with the BIA method (25). As a method that introduces an electrical current and assesses its transmission through the body, it is very important to monitor the levels of body water (9). Hydration status has also been proposed as a variable to control for when performing DXA body composition assessment (70). This could be explained by the water component that is present in the different body tissues, not only in muscle mass but also in tissues containing the fat component (22, 70). However, in the present research, hydration status did not have high impact. This could be because in the present study, we controlled for the main factors that could affect hydration status, such as the practice of physical exercise, the consumption of products with diuretic properties, eating heavy meals, having an injury, taking hormonal or corticosteroid treatment, or the timing of the menstrual cycle in the case of women (71–74). Therefore, it is possible that the hydration conditions of the present sample were fairly standardized, which would have resulted in the hydration status not influencing the agreement between methods and formulas. These results should be tested in future research, where these factors that could affect hydration are not controlled for.

Despite the above, a noteworthy result of the present investigation was that, in case DXA assessment for fat mass cannot be used, Carter’s anthropometry formula could be a good alternative to evaluate fat mass of individual subjects in general sample. This formula includes skinfolds of the upper limb, trunk and lower limb for estimation of fat mass, which approximates the subject’s overall fat mass, as is done by DXA scanning (27). An alternative to the above could be the Forsyth formula for the percentage of fat mass in the general population. It is important to mention that these formulas include active persons in their validation (52). Therefore, it would be important to assess whether these alternatives remain appropriate in a population of different age, activity levels or with different pathologies considering that these factors have been shown to affect the validity of the formulas (35). Furthermore, Evans’s anthropometry formula was shown as a good alternative to evaluate fat mass of individual subjects in female sample. It is important to mention that this formula requires few variables (triceps, abdominal and thigh skinfold) which makes it a quick formula to obtain. In addition, some previous studies also found this better concordance of the Evans formula in young females in comparison with other anthropometry formulas (75).

Furthermore, if a group analysis is desired, BIA could be a good alternative in the case of women. Previous studies have suggested that the BIA, without being a valid method for the assessment of individual subjects, can be a valid method for the assessment of groups (76). However, the influence of sex on this issue has not been analyzed so far. The present investigation demonstrates that BIA is a good alternative for the assessment of women’s groups only, provided that some of the factors that could most influence the assessment of fat mass with BIA, such as the time of the menstrual cycle at which the assessment is performed (77), food or fluid intake (78) or the practice of physical exercise (79) are controlled.

This study provides practical applications for nutritionists, trainers, physiotherapists, and any health professional who is interested in the fat mass of their patient and/or athlete. The fact that DXA, anthropometry and BIA are not interchangeable, and neither anthropometry and BIA are valid with respect to DXA in general terms, forces the practitioner to always use the same method and formula to monitor the changes in fat mass of their patients/users over time. On the other hand, when seeking to compare an athlete with fat mass references in their discipline or a subject with the fat mass references for a non-communicable disease, the practitioner must ensure that the same formula and method used in the references study are used. In addition, due to the important influence found with respect to sex, it is essential to have different working protocols and to use different methods depending on the sex of the person to be assessed.

This study also had some limitations. On the one hand, we used formulas that had not been validated only in the population analysed. However, this is often a standard practice in both clinical and research settings, a decision was made to apply them to discover what was occurring in the analysis. Another limitation was the use of a regression formulas to “convert” Kerr’s formula dealing with “adipose tissue” to “fat mass.” This was done to determine whether converting all methods and formulas to the same component would maintain the differences. Furthermore, with respect to BIA, it was done in a standing position with a model that did not give all the electrical properties in its report. Therefore, it has not been possible to calculate fat mass with different bioelectrical formulas for bioimpedance in this work, as was done for anthropometry (21) and as has been proposed that this should be done in previous studies with BIA (21). Finally, in the present investigation, the assessment of hydration status was evaluated to analyse its influence on the results found, but was not manipulated. Therefore, future research should manipulate hydration status to analyse its influence on the agreement and validity of anthropometry and BIA with respect to DXA (78, 80).

In conclusion, the formulas for fat mass assessment with anthropometry and BIA may not be valid with respect to the values reported with DXA, with the exception of Carter’s and Forsyth’s anthropometry formulas for general sample, and Evans’s anthropometry formula for female sample. BIA could also be an alternative if what is needed is to assess fat mass in women as a group.

## Data availability statement

The raw data supporting the conclusions of this article will be made available by the authors, without undue reservation.

## Ethics statement

The studies involving humans were approved by the Ethics Committee from the Catholic University San Antonio of Murcia (Murcia, Spain) reviewed and authorized the protocol designed for data collection, considering the World Medical Association Code (CE062103). The studies were conducted in accordance with the local legislation and institutional requirements. The participants provided their written informed consent to participate in this study.

## Author contributions

MM-C: Writing – review & editing, Writing – original draft, Methodology, Investigation, Formal analysis, Data curation. MA-S: Writing – review & editing, Writing – original draft, Methodology, Investigation, Formal analysis, Data curation. RV-C: Writing – review & editing, Writing – original draft, Supervision, Project administration, Investigation, Funding acquisition, Formal analysis, Data curation, Conceptualization. NB: Writing – review & editing, Writing – original draft, Methodology, Investigation. FE-R: Writing – review & editing, Writing – original draft, Supervision, Resources, Project administration, Funding acquisition, Conceptualization.

## Supplementary material

SUPPLEMENTARY FIGURE S1 Graphical representation of the means and standard deviations of fat mass in kg for the general sample.

SUPPLEMENTARY FIGURE S2 Graphical representation of the means and standard deviations of fat mass in percentage for the general sample.

SUPPLEMENTARY FIGURE S3 Graphical representation of the means and standard deviations of fat mass in kg in males.

SUPPLEMENTARY FIGURE S4 Graphical representation of the means and standard deviations of fat mass in percentage in males.

SUPPLEMENTARY FIGURE S5 Graphical representation of the means and standard deviations of fat mass in kg in females.

SUPPLEMENTARY FIGURE S6 Graphical representation of the means and standard deviations of fat mass in percentage in females.
