# Corrigendum: Validity and agreement between dual-energy X-ray absorptiometry, anthropometry and bioelectrical impedance in the estimation of fat mass in young adults

**DOI:** 10.3389/fnut.2024.1488063

**Published:** 2024-10-21

**Authors:** Malek Mecherques-Carini, Mario Albaladejo-Saura, Raquel Vaquero-Cristóbal, Nicolás Baglietto, Francisco Esparza-Ros

**Affiliations:** ^1^International Kinanthropometry Chair, UCAM Universidad Católica San Antonio de Murcia, Murcia, Spain; ^2^Department of Physical Activity and Sport Sciences, Faculty of Sport Sciences, University of Murcia, San Javier, Spain

**Keywords:** body composition, fat mass, anthropometry, bioelectrical impedance analysis, dual-energy X-ray absorptiometry

In the published article, there was an error in **Material and methods**, *Design*, paragraph 1. The following sentence was erroneously added “The statistical power of the study was calculated for the sample indicated for the physical activity and AMD variables.”

The paragraph previously read as:

“The present research followed a descriptive, cross-sectional design. The sample recruitment was non-probabilistic by convenience. The calculation used to establish the minimum sample size was performed with Rstudio 3.15.0 software (Rstudio Inc., Boston, MA, United States). The significance level was set at α = 0.05. The standard deviation (SD) for the total sample was set based on previous studies on the variables of fat mass percentage (SD = 5.19) (8). This methodology for sample size calculation has been used in previous research (42). Thus, the minimum sample size was 265 subjects, assuming an error (*d*) of 0.62% for fat mass percentage and for a 99% confidence interval (CI). Considering that acceptable statistical power is greater than 0.80 (43). The statistical power of the study was calculated for the sample indicated for the physical activity and AMD variables. The calculated statistical power was 0.96, which is high.”

The corrected paragraph should read as:

“The present research followed a descriptive, cross-sectional design. The sample recruitment was non-probabilistic by convenience. The calculation used to establish the minimum sample size was performed with Rstudio 3.15.0 software (Rstudio Inc., Boston, MA, United States). The significance level was set at α = 0.05. The standard deviation (SD) for the total sample was set based on previous studies on the variables of fat mass percentage (SD = 5.19) (8). This methodology for sample size calculation has been used in previous research (42). Thus, the minimum sample size was 265 subjects, assuming an error (*d*) of 0.62% for fat mass percentage and for a 99% confidence interval (CI). Considering that acceptable statistical power is greater than 0.80 (43). The calculated statistical power was 0.96, which is high.”

The authors apologize for this error and state that this does not change the scientific conclusions of the article in any way. The original article has been updated.

