# Supplementary figures and images for: Validity and agreement between dual-energy X-ray absorptiometry, anthropometry and bioelectrical impedance in the estimation of fat mass in young adults
Source: Front Nutr. 2024 Jun 11;11:1421950. doi: 10.3389/fnut.2024.1421950 (PMC11198126; doi:10.3389/fnut.2024.1421950)

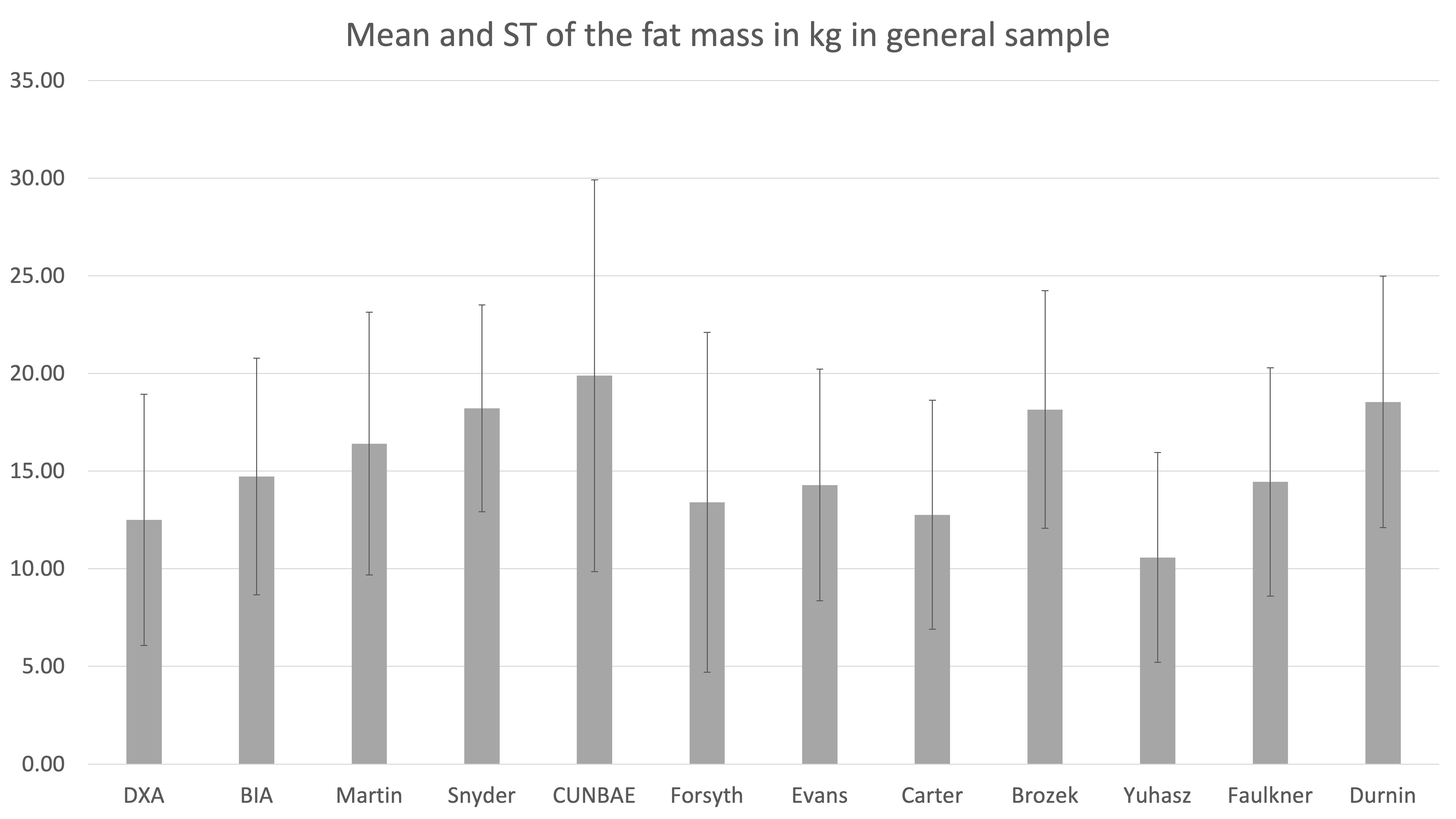

Supplement: SUPPLEMENTARY FIGURE S1 — Graphical representation of the means and standard deviations of fat mass in kg for the general sample. [file Image_1.JPEG]

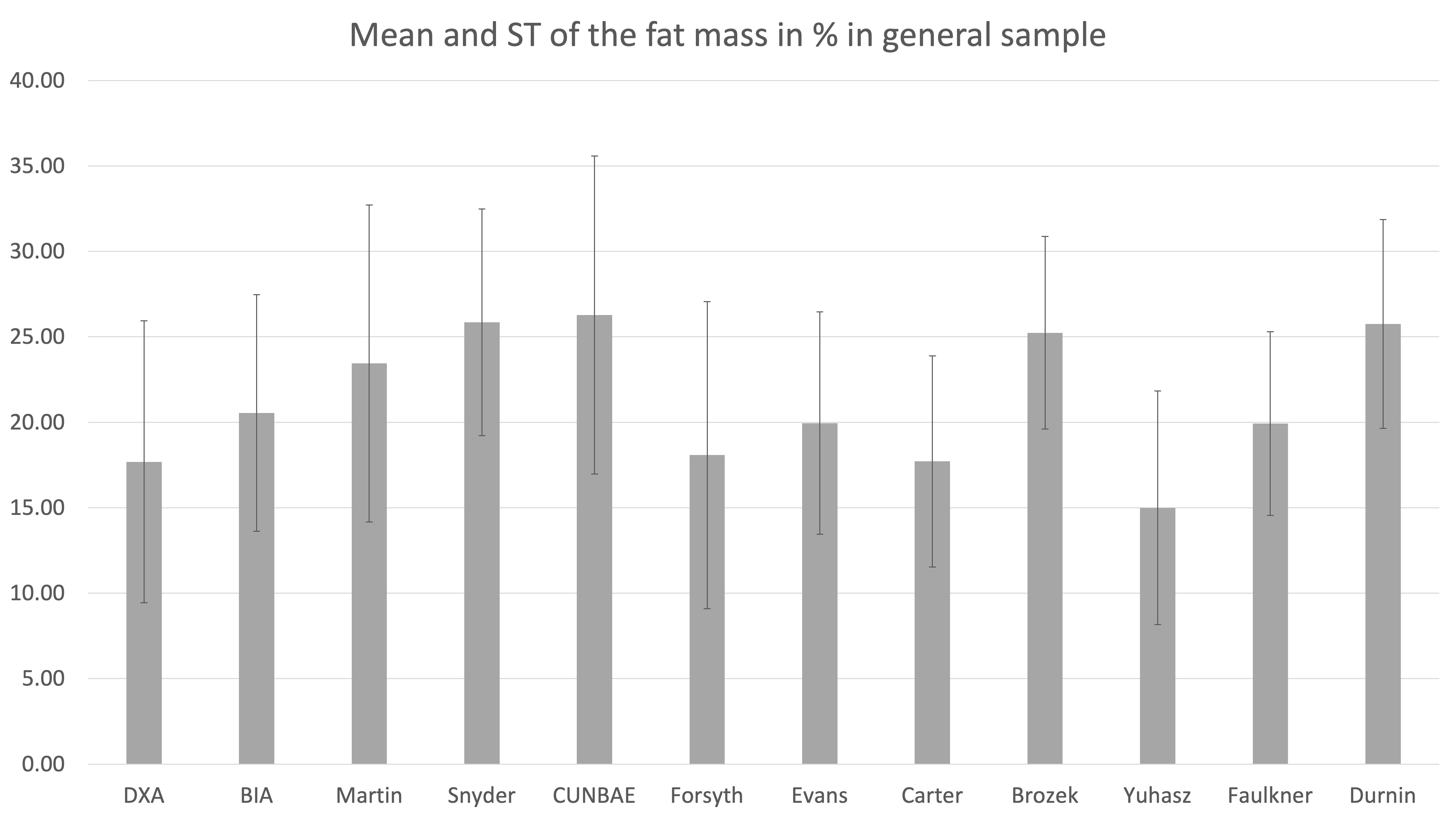

Supplement: SUPPLEMENTARY FIGURE S2 — Graphical representation of the means and standard deviations of fat mass in percentage for the general sample. [file Image_2.JPEG]

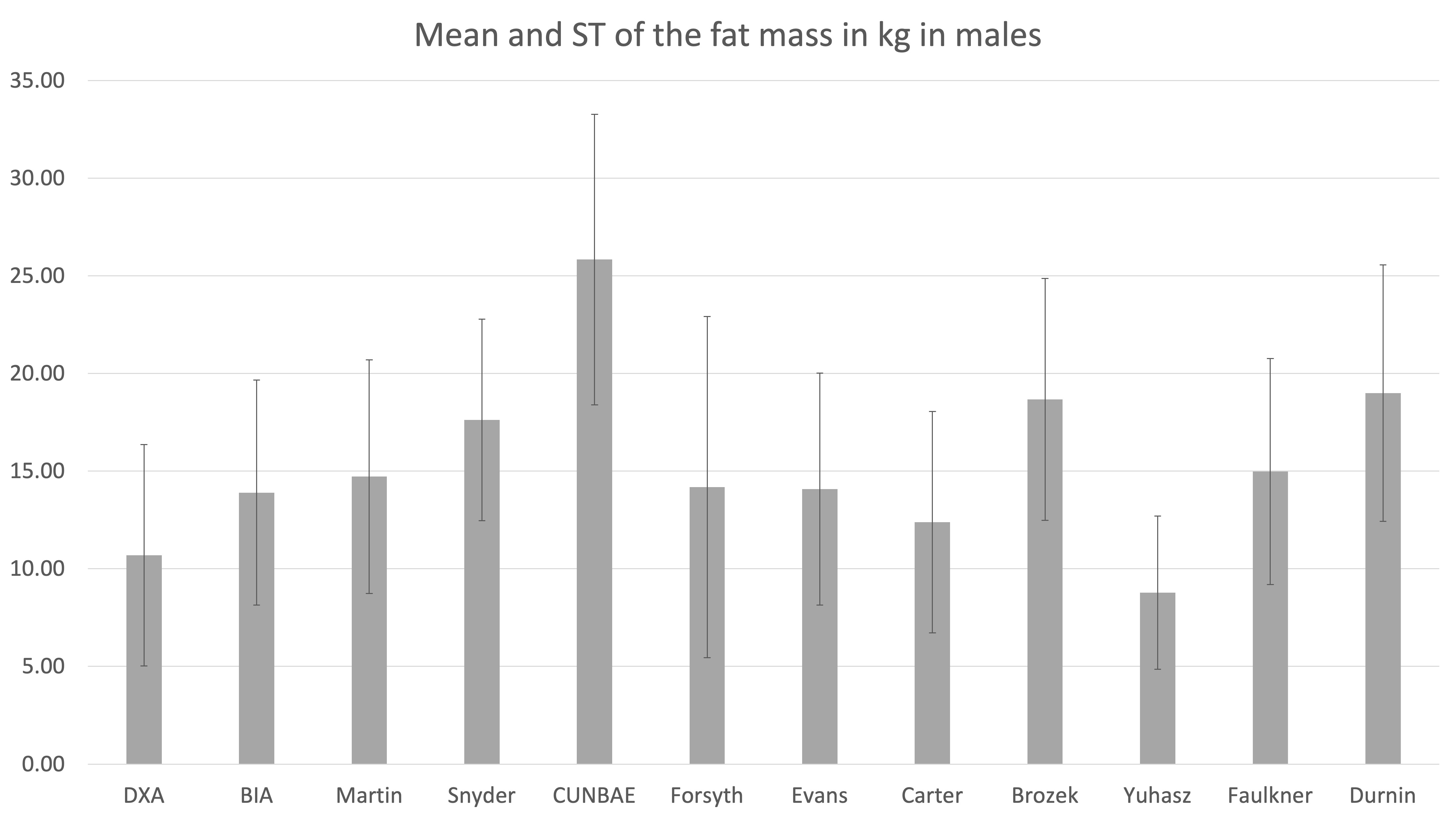

Supplement: SUPPLEMENTARY FIGURE S3 — Graphical representation of the means and standard deviations of fat mass in kg in males. [file Image_3.JPEG]

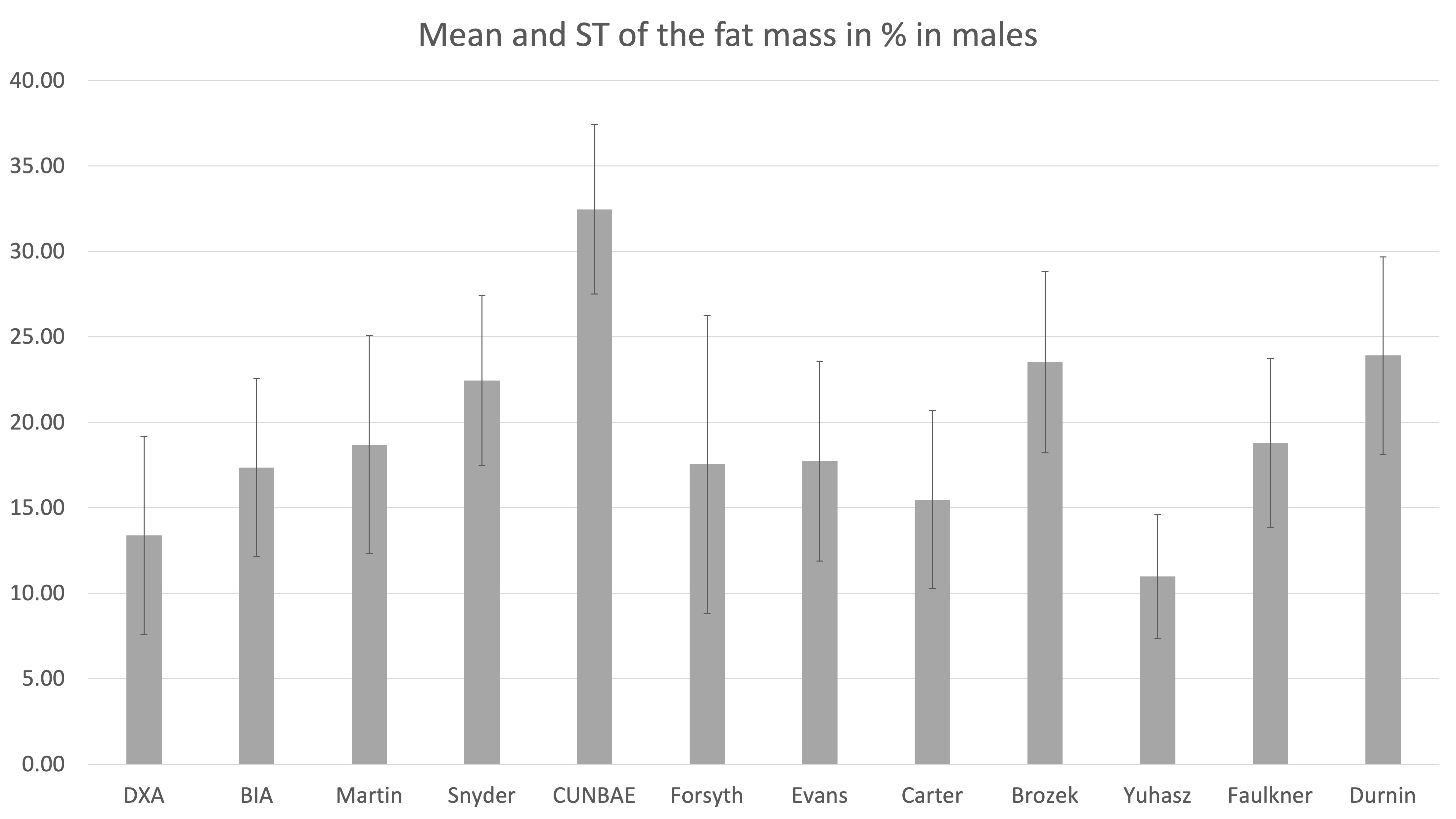

Supplement: SUPPLEMENTARY FIGURE S4 — Graphical representation of the means and standard deviations of fat mass in percentage in males. [file Image_4.JPEG]

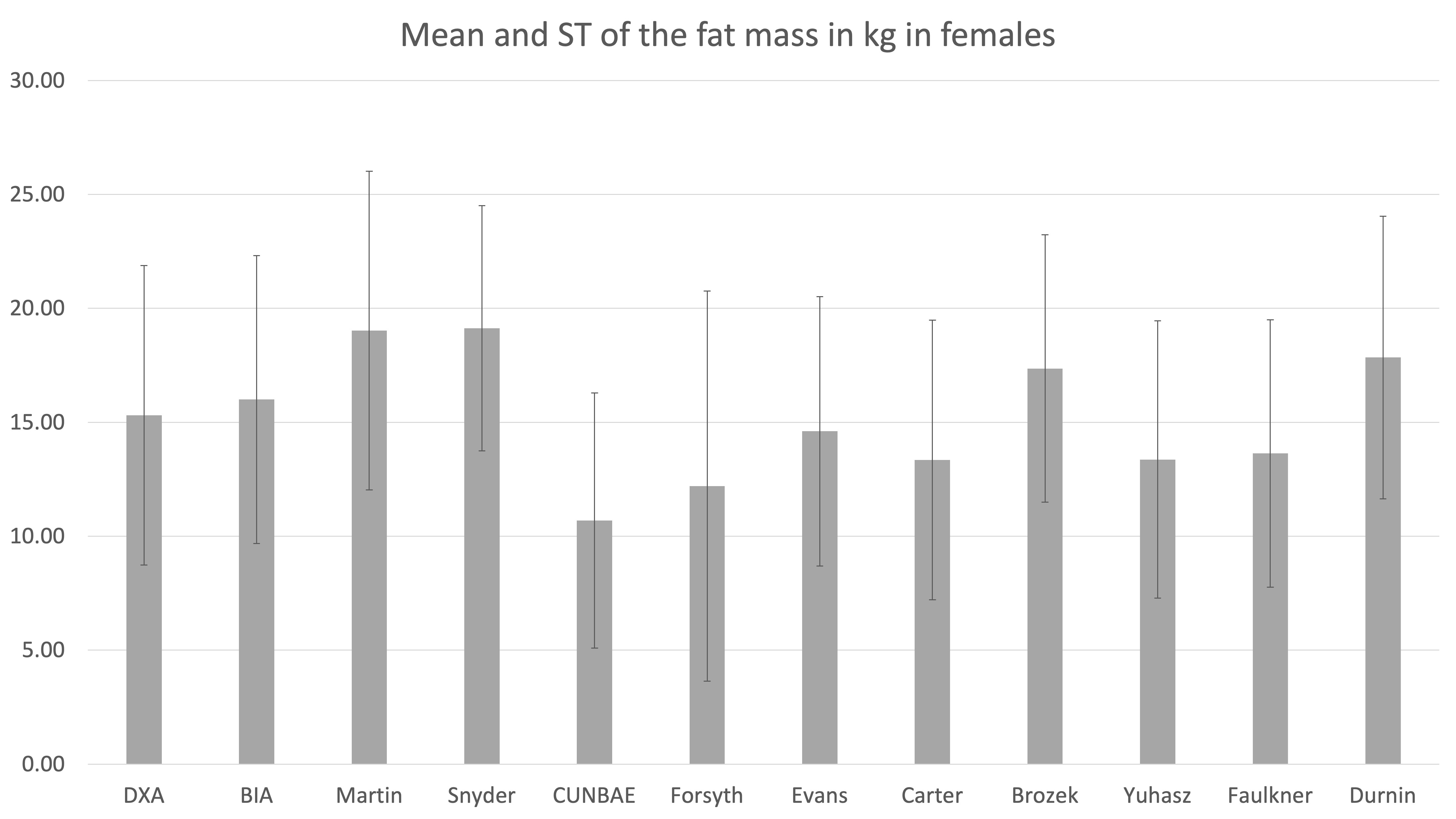

Supplement: SUPPLEMENTARY FIGURE S5 — Graphical representation of the means and standard deviations of fat mass in kg in females. [file Image_5.JPEG]

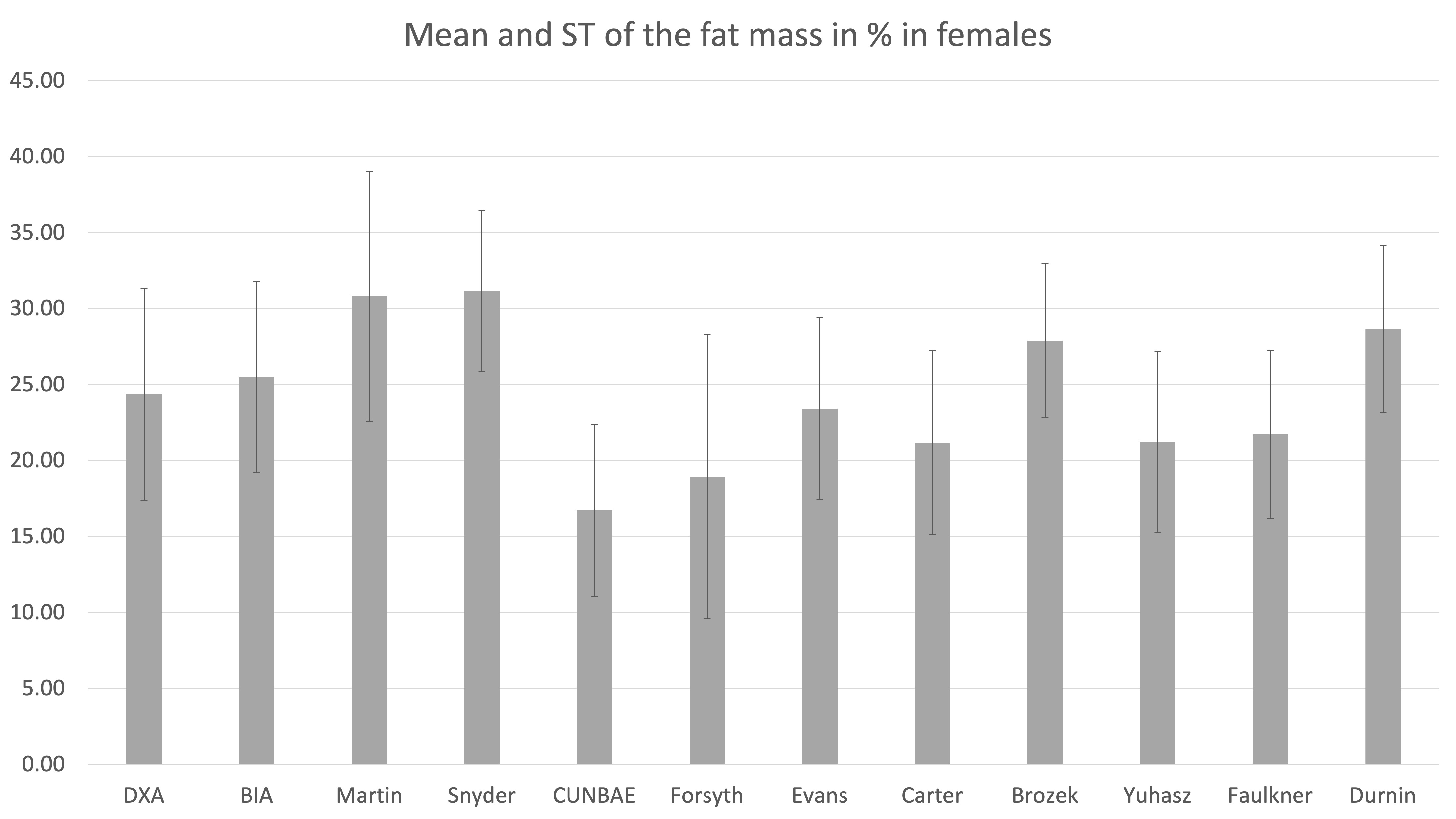

Supplement: SUPPLEMENTARY FIGURE S6 — Graphical representation of the means and standard deviations of fat mass in percentage in females. [file Image_6.JPEG]
